# GC–MS‐Based Phytochemical Profiling and Integrated Evaluation of Antioxidant, Antibacterial, and *In Silico* Studies of Kumaun Himalayan *Strobilanthes* Species

**DOI:** 10.1002/cbdv.71680

**Published:** 2026-09-04

**Authors:** Sakshi Negi, Ravendra Kumar, Vaishnavi Rawat, Pooja Bargali, Aashish Kumar Sagar, Stefania Garzoli, Riddhiman Lahiri, Shilpi Rawat, Avdhesh Kumar, Manmohan Singh Rawat, Jolanta Maslowiecka, Valery A. Isidorov, Dharmendra S. Rawat

**Affiliations:** ^1^ Department of Chemistry, College of Basic Sciences and Humanities G.B. Pant University of Agriculture and Technology U.S. Nagar Uttarakhand India; ^2^ Department of Chemistry and Technologies of Drug Sapienza University Rome Italy; ^3^ Department of Plant Pathology, College of Agriculture G.B. Pant University of Agriculture and Technology U.S. Nagar Uttarakhand India; ^4^ Department of Aquaculture, College of Fisheries G.B. Pant University of Agriculture and Technology U.S. Nagar Uttarakhand India; ^5^ Uttarakhand State Council For Science & Technology (UCOST) Dehradun Uttarakhand India; ^6^ Institute of Forest Sciences, Bialystok Technology University Bialystok Poland; ^7^ Department of Biological Sciences, College of Basic Sciences and Humanities G.B. Pant University of Agriculture and Technology U.S. Nagar Uttarakhand India

**Keywords:** Acanthaceae, chemical investigation, *Aeromonas salmonicida*, antioxidants, *Edwardsiella tarda*, *Strobilanthes*

## Abstract

Five *Strobilanthes* species indigenous to the Kumaun Himalayan region of Uttarakhand, India, have been historically employed in traditional medicine to treat bacterial infections, inflammatory conditions, and respiratory ailments, but remain largely unexplored for aquaculture disease management applications. Dichloromethane extracts of *S. glutinosa* (SGDE), *S. pentastemonoides* (SPDE), *S. angustifrons* (SADE), *S. attenuata* (SATDE) and *S. tomentosa* (STDE) were comprehensively analysed by GC‐MS for phytochemical profiling, with antioxidant activity quantified using DPPH radical scavenging and ferrozine‐based metal chelating assays, and antibacterial efficacy evaluated against *Aeromonas salmonicida* and *Edwardsiella tarda* using disc diffusion methodology. Pearson correlation analysis, molecular docking, and ADMET computational modelling were performed to elucidate structure‐activity relationships and pharmacokinetic properties. GC‐MS analysis identified 42‐59 phytochemical constituents per species, with sterols and triterpenes constituting the predominant class. STDE demonstrated superior DPPH radical scavenging activity, while SATDE exhibited optimal metal chelating potential. SGDE and STDE displayed the good antibacterial activities against both pathogens. Pearson correlation analysis identified n‐tritriacontane and sclareol associated with antimicrobial activity, while molecular docking revealed superior binding affinities for β‐amirone to DNA topoisomerase I and β‐amyrin. ADMET assessment identified sclareol, stigmasterol, and 24‐epicampesterol as optimal candidates with favorable.

## Graphical abstract

Himalayan *Strobilanthes* species: phytochemical profiling, bioactivities, and In silico Studies.

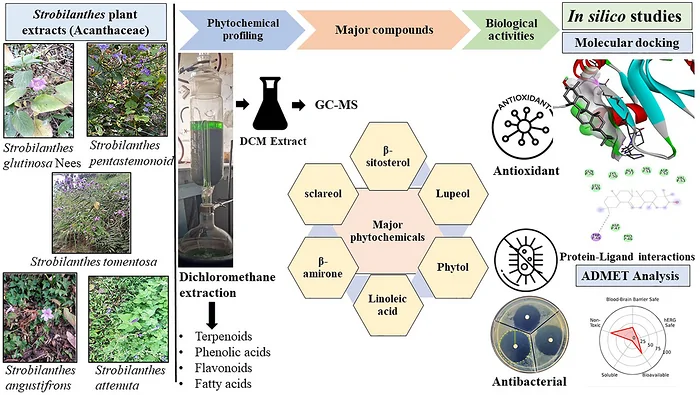

## 1 Introduction

Aquaculture has emerged as the fastest‐growing food production sector globally, now surpassing capture fisheries as the primary source of seafood for human consumption. Projected to supply nearly half of the world's edible fish by 2030, this sector plays a critical role in global food security, contributing approximately 16% of global animal protein intake and providing essential omega‐3 fatty acids, minerals and micronutrients to populations worldwide [1, 2, 3]. Bacterial infections are the foremost disease challenge in aquaculture, particularly in warm‐water systems, resulting in billions of dollars in annual global losses [4, 5]. Antibiotic residues in aquaculture products and effluents pose serious risks to human health and environmental integrity, including the emergence of multidrug‐resistant pathogens and disruption of aquatic microbial communities [6, 7]. These challenges underscore the urgent need for sustainable, efficacious alternatives to conventional antibiotics. Phytochemicals, bioactive secondary metabolites derived from medicinal plants have emerged as promising candidates for disease management in aquaculture due to their broad‐spectrum antimicrobial activity, immunomodulatory properties and environmental safety [8, 9, 10]. Plants synthesize structurally diverse secondary metabolites, including terpenoids, phenolic compounds, alkaloids, tannins, and lignins through specialized biosynthetic pathways [11, 12]. These compounds function as adaptive chemical mediators in ecological interactions, conferring protection against herbivory, pathogen attack and abiotic stresses, while exhibiting potent antioxidant, anti‐inflammatory, and antimicrobial activities in biological systems [13, 14, 15]. Phytochemicals have demonstrated efficacy against key bacterial pathogens, including *Aeromonas* spp., *Edwardsiella tarda* and *Vibrio* spp., through both direct antimicrobial action and enhancement of host innate immunity [16, 17, 18].

The *Acanthaceae* family, comprising over 4000 species is recognized for its rich ethnopharmacological heritage and diverse phytochemical profile [19, 20, 21]. Within this family, the genus *Strobilanthes*, encompassing approximately 350 species distributed across tropical and subtropical Asia, is particularly notable for its abundance of bioactive compounds, including diterpenoids, flavonoids, phytosterols, phenolic acids, alkaloids and triterpenes [22, 23, 24].

Various *Strobilanthes* species are widely used in traditional medicine system and ethnopharmacological practices for treating ailments such as diabetes, inflammation, respiratory disorders, infections, rheumatism, and kidney diseases, while some species also serve ornamental and dye‐producing purposes [25]. *Strobilanthes cusia* has a documented therapeutic history spanning nearly a millennium in traditional Chinese medicine, with applications in treating febrile conditions, influenza, mumps, and epidemic encephalitis [26]. S*trobilanthes* species exhibit a broad spectrum of pharmacological activities, including antioxidant, anti‐inflammatory, antimicrobial, antiviral, anticancer, antidiabetic, and wound‐healing effects, largely attributed to their rich content of flavonoids, phenolics, terpenoids, alkaloids, and glycosides. These bioactivities support their traditional uses [25].

Despite the traditional applications and demonstrated pharmacological potential of these *Strobilanthes* species, their application in aquaculture remains largely unexplored, particularly regarding their efficacy against fish pathogens, especially *E. tarda* and *A. salmonicida*. Previous research on *Strobilanthes* species focused on the phytochemical composition and biological potential of essential oils native to the Kumaun Himalayan region of Uttarakhand, India [27], leaving significant gaps in comparative evaluations. Furthermore, the bioactive constituents responsible for the observed biological activities have not been systematically correlated with their pharmacological potential.

The present study focuses on a comparative study of dichloromethane extracts of five *Strobilanthes* species to comprehensively investigate their phytochemical profiles, antioxidant capacities and antibacterial activities against *E. tarda* and *A. salmonicida*. This research aims to advance the understanding of *Strobilanthes* species as sustainable, natural alternatives for disease management in aquaculture, contributing to environmentally responsible therapeutic strategies that address antimicrobial resistance and sustainability in global aquaculture production.

## 2 Materials and Methods

### 2.1 Collection and Identification of Plant Material

Specimens of various *Strobilanthes* species were collected during their blooming phase from five distinct locations across the Kumaun region of Uttarakhand, India (Table 1). All specimens were authenticated by Dr. D. S. Rawat (Department of Biological Sciences, CBSH, G.B.P.U.A.&T., Pantnagar), The voucher specimen for different identified *Strobilanthes* species viz., *Strobilanthes glutinosa* Nees, *Strobilanthes pentastemonoid* (Nees) T. Anderson, *Strobilanthes angustifrons* C. B. Clarke, *Strobilanthes attenuta* (Wall. ex Nees) Jacq. Ex Nees, *Strobilanthes tomentosa* (Nees) J.R.I.Wood., with voucher numbers GBPUH‐ 1041, GBPUH‐1030, GBPUH‐1031, GBPUH‐1029, and GBPUH‐1042 were deposited at the herbarium of Department of Biological Sciences, for future references.

**TABLE 1:** Collection details and voucher information of the five selected *Strobilanthes* species.

S. No.	Plant species	Sample code	Collection site	Date of collection	Voucher specimen
1.	*Strobilanthes glutinosa* Nees	SGDE	Kill, Pithoragarh (Uttarakhand)	19‐Jan‐2019	GBPUH‐ 1041
2.	*Strobilanthes pentastemonoid* (Nees) T. Anderson	SPDE	Sirkuch, Pithoragarh (Uttarakhand)	18‐Oct‐2021	GBPUH‐1030
3.	*Strobilanthes angustifrons* C. B. Clarke	SADE	Garur, Bageshwar (Uttarakhand)	29‐Jan‐2021	GBPUH‐1031
4.	*Strobilanthes attenuta* (Wall. ex Nees) Jacq. ex Nees	SATDE	Saktipur, Champawat (Uttarakhand)	20‐Aug‐2021	GBPUH‐1029
5.	*Strobilanthes tomentosa* (Nees) J.R.I.Wood	STDE	Kainchi dham, Nainital (Uttarakhand)	20‐Sept‐ 2021	GBPUH‐1042

### 2.2 Extract Preparation

Fresh leaves, twigs and flowers of five *Strobilanthes* species were subjected to hydrodistillation using a Clevenger‐type apparatus for 3 hours to obtain essential oils (EOs). The volatile fractions were discarded to eliminate analytical interference from EO constituents [27, 28]. The residual plant material was shade‐dried, finely powdered and extracted with dichloromethane (DCM) using the cold percolation method for 72–96 hours [29]. DCM was selected based on its mid‐polarity index (3.1) and efficiency in recovering a broad range of bioactive compounds, including terpenoids and phenolics [30]. The extracts were vacuum‐filtered through Whatman No. 1 paper, combined and concentrated under reduced pressure at 50°C to obtain crude DCM extracts. The extraction yield was calculated based on the dry weight of the plant material.

$$\mathrm{Percent}\;\mathrm{yield}\left(\%\right)=\frac{\mathrm{weight}\;\mathrm{of}\;\mathrm{dried}\;\mathrm{extract}\left(\mathrm{gm}\right)}{\mathrm{weight}\;\mathrm{of}\;\mathrm{plant}\;\mathrm{material}\left(\mathrm{gm}\right)}\times 100$$

### 2.3 Phytochemical Analysis by GC‐MS

Gas chromatography‐mass spectrometry analysis was performed using an Agilent 7890A GC equipped with a 5975C VL MSD detector and HP‐5MS capillary column (30 m × 0.25 mm × 0.25 µm). Helium was used as carrier gas (1.0 mL/min). Sample injection (1 µL) was in split mode (1:10) at 250°C. Oven temperature was programmed from 50°C to 325°C at 3°C/min, held for 10 min. Mass spectra were acquired in electron impact mode (70 eV). Retention indices were calculated using a standard mixture of C_10_‐C_40_ n‐alkanes in hexane. Following integration, the fraction of separated components in the total ion current was computed using the equation provided below:

$$RI^{\mathrm{Cal}}=100\left[n+(RT_{x}-RT_{n}\right)/\left(RT_{n+1}-RT_{n}\right)]$$

where RT_x_ = retention time of the analyte, RT_n_ = retention time of n‐alkane, eluting directly before the analyte.

Compounds were identified by comparing mass spectra with NIST and Adams' libraries [31]; identification required MS similarity ≥85% and RI deviation ≤ ± 10 units.

### 2.4 Antioxidant Activity

#### 2.4.1 DPPH Radical Scavenging Assay

Extracts (0.05‐5 mg/mL in methanol) were mixed with 0.1 mM DPPH solution (3:100 µL ratio) and incubated in darkness for 30 min. Absorbance was measured at 517 nm with methanol serving as the blank and Butylated hydroxytoluene as the positive control [32, 33, 34]. Radical scavenging activity (%) was calculated as:

$$\mathrm{IC}\%=\frac{\mathrm{Ao}-\mathrm{At}}{\mathrm{Ao}}\times 100$$

where Ao = Absorbance value of Control sample, At = Absorbance value of test sample, IC = Inhibitory concentration.

#### 2.4.2 Metal Chelating Assay

Metal chelating activity of Fe^2+^ by the DCM extracts was determined using a modified ferrozine‐based spectrophotometric method, as described previously [34] to accommodate DCM‐soluble samples. Briefly, 100 µL of plant extract (10‐50 µg/mL, in methanol) was incubated with 100 µL 2 mM FeCl_2_ · 4H_2_O and 200 µL 5 mM ferrozine. The reaction volume was adjusted to 5.0 mL with methanol and incubated in the dark at 25°C for 10 min to form the Fe^2+^‐ferrozine complex. Absorbance was measured at 562 nm using a UV–vis spectrophotometer, with methanol as a blank. Disodium EDTA served as standard. Negative controls (all reagents but extract), sample blanks (extract and methanol only) and a positive control (EDTA) were run in parallel. The percentage of metal chelating activity was calculated using the formula:

$$\mathrm{IC}\%=\frac{\mathrm{Ao}-\mathrm{At}}{\mathrm{Ao}}\times 100$$

where Ao = Absorbance of control, At = Absorbance of sample, IC = Inhibitory concentration.

All samples were assayed in triplicate. IC_50_ values were calculated by nonlinear regression analysis of per cent inhibition versus extract concentration for both DPPH radical scavenging and metal chelating assays. This approach was employed due to its suitability for reliable quantification of antioxidant and metal chelating activities in plant‐derived extracts, including those solubilised in dichloromethane.

### 2.5 Antibacterial Activity

Antibacterial activity was assessed against *Edwardsiella tarda* and *Aeromonas salmonicida* using the disc diffusion method according to CLSI M02‐A12 guidelines. Bacterial strains were cultured on Nutrient Agar (SRL, Ref: 63971) and standardized to a 0.5 McFarland standard (∼1.5 × 10^8^ CFU/mL) [35, 36]. Plant extracts were prepared in 10% Tween‐20 at concentrations of 100, 200, and 300 mg/mL; 10% Tween‐20 served as a negative control and streptomycin (1 mg/mL) as a positive control. Nutrient Agar plates were inoculated with standardized bacterial suspensions and sterile 6‐mm discs impregnated with 10 µL of test samples were placed on the agar surface. Plates were incubated at 37°C for 24 h, and inhibition zone diameters were measured in millimetres (mm).

### 2.6 Molecular Docking

Molecular docking studies were performed to evaluate the binding affinities and interaction profiles of major phytocompounds, which were selected on the basis of their high abundance with bacterial proteins 30S ribosomal subunit (PDB ID: 4DR3) and DNA topoisomerase I (PDB ID: 1CY8). Three‐dimensional protein structures were retrieved from the RCSB Protein Data Bank (https://www.rcsb.org/). Phytocompounds were sourced from PubChem database in SDF format. Streptomycin (PubChem CID: 19649) and ciprofloxacin (PubChem CID: 2764) were used as reference ligands for 4DR3 and 1CY8, respectively, reflecting their established clinical efficacy. Docking simulations were conducted using CB‐Dock2 (https://cadd.labshare.cn/cb‐dock2/php/index.php.), employing cavity‐detection algorithms for enhanced accuracy. Receptor‐ligand interactions, including hydrogen bonding and hydrophobic contacts, were visualized and analyzed using Biovia Discovery Studio 2023 Client to generate both 2D and 3D interaction diagrams [32, 37].

### 2.7 ADMET Prediction

Absorption, Distribution, Metabolism, Excretion, Toxicity (ADMET) analysis was performed to predict the pharmacokinetic properties and safety profiles of major phytocompounds with high abundance. Chemical structures were retrieved from the PubChem database in SMILES format and subjected to computational evaluation. ADMET parameters were systematically analyzed using the ADMET AI platform (https://admet.ai.greenstonebio.com/). Toxicity profiling was additionally conducted using ProTox3.0 (https://tox.charite.de/protox3/index.php?site=compound_input) to generate toxicity radar charts for comparative risk assessment within chemical classes. This integrated approach enabled comprehensive evaluation of the compounds' pharmacokinetic behavior and safety profiles, supporting their potential utility as sustainable alternatives to synthetic agents.

### 2.8 Statistical Analysis

All in vitro bioassays were performed in triplicate and data were expressed as mean ± standard error. Statistical analysis was conducted using one‐way and two‐way analysis of variance (ANOVA) in OriginPro 2024 (version 10.0.0.154). Significant differences between treatment means were determined at *p* ≤ 0.05 using Tukey's post hoc test. Chemometric analyses, including hierarchical cluster analysis (HCA) and Pearson's correlation coefficient calculations, were performed to visualize bioactivity patterns and dose‐response relationships.

## 3 Results and Discussion

### 3.1 Chemical Composition

**FIGURE 1:**
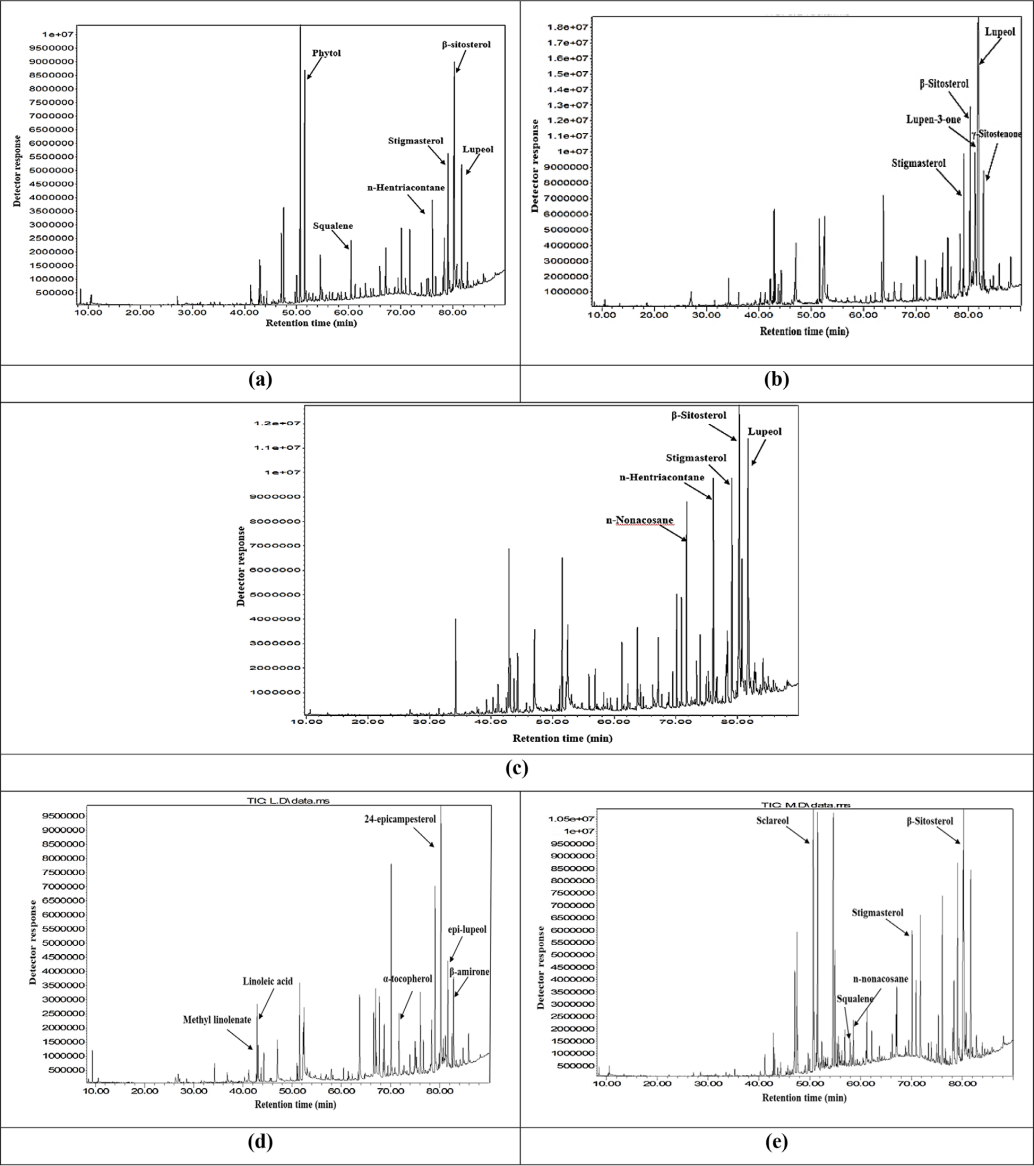
TIC Profiles of Five *Strobilanthes* Species: (a) SGDE, (b) SPDE, (c) SADE, (d) SATDE, and (e) STDE.

**FIGURE 2:**
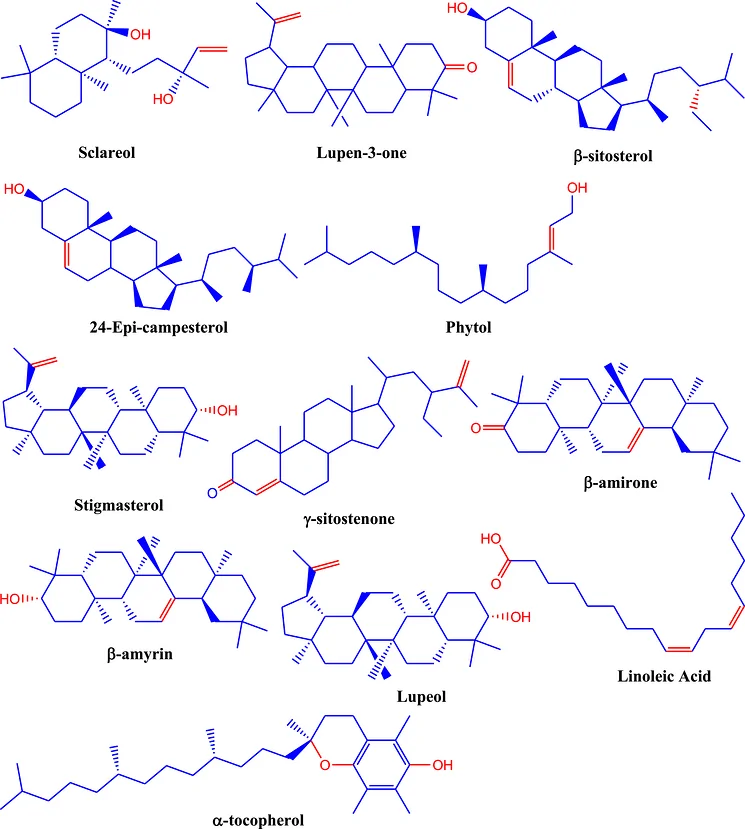
Structures of major components of Extract of SGDE, SPDE, SADE, SATDE, and STDE.

Comprehensive phytochemical profiling by gas chromatography‐mass spectrometry (GC‐MS) of five *Strobilanthes* species revealed distinct yet interconnected chemical signatures. *S. glutinosa* (SGDE) yielded 42 identified compounds representing 68.1% extract composition, *S. penstemonoid* (SPDE) yielded 59 compounds (80.43%), *S. angustifrons* (SADE) yielded 53 compounds (86.38%), *S. attenuata* (SATDE) yielded 37 compounds (62.72%), and *S. tomentosa* (STDE) yielded 48 compounds (71.19%). Only the major compounds with high abundance are presented in Table 2, while the complete dataset of identified major and minor compounds are provided as Supporting Information S1. Sterols and triterpenes constituted the predominant phytochemical class across all species, with varying proportional contributions: SPDE (55.48%), SADE (43.18%), STDE (25.89%), SGDE (27.52%), and SATDE (27.64%). β‐Sitosterol and lupeol emerged as the most abundant and universally distributed marker compounds across all five species. β‐Sitosterol concentrations varied considerably among species, ranging from 1.48% in SATDE to 11.29% in SADE, while lupeol abundances ranged from 1.34% (SATDE) to 22.63% (SPDE), with SPDE demonstrating the highest lupeol concentration. Both possess documented anticancer, anthelminthic, antimutagenic and hypocholesterolemic activities [38, 39, 40, 41, 42] along with antioxidant, antiproliferative and anti‐inflammatory properties [43, 44, 45], positioning these ubiquitous constituents as primary contributors to the purported bioactivity across all five species. Additional triterpenes including β‐amyrin (1.32%–4.44%), stigmasterol (0.80%–6.92%), and stigmastane‐3,6‐dione (0.34%–1.47%) were detected in all species, establishing a conserved triterpene framework characteristic of the genus. Species‐specific triterpene profiles distinguished individual taxa: SPDE was uniquely enriched in lupen‐3‐one (7.44%) and γ‐sitostenone (4.24%), SATDE exhibited elevated β‐amirone (5.34%) and epi‐lupeol (4.35%) which enhance antioxidant and anti‐inflammatory profiles [46, 47, 48] and STDE was characterized by pronounced sclareol (10.72%), a labdane‐type diterpene, which exhibits multimodal bioactivity encompassing anticancer, antibacterial, neuroprotective, anti‐inflammatory, antihypertensive, and antidiabetic properties [49, 50, 51, 52, 53]. Long‐chain aliphatic hydrocarbons represented the second most substantial phytochemical category, contributing from 10.86% (SATDE) to 23.78% (SADE) of total composition. The consistent identification of n‐hentriacontane, n‐nonacosane, and n‐tritriacontane across all five species, despite varying proportional abundances, indicates their role in conferring antimicrobial and anti‐inflammatory activities [54, 55, 56]. SATDE and SADE accumulated elevated linoleic acid and methyl linolenate (7.44% and 4.66%, respectively), whereas SGDE showed minimal aliphatic acid content (3.19%), with the enriched linoleic and linolenic acids conferring antioxidant, anti‐inflammatory, neuroprotective, anticancer and antiosteoporotic activities complementary to their sterol‐triterpene core [57, 58, 59, 60]. Phytol, a diterpenoid alcohol consistently identified in all species (1.86%–8.12%), correlates with antimicrobial, cytotoxic, antioxidant, and anticancer effects [61, 62, 63, 64]. Squalene, an important sesquiterpene precursor, was universally detected at concentrations ranging from 1.01% (SPDE) to 2.57% (SATDE) contributes antioxidant, antitumor and antibacterial properties [65, 66, 67]. Neophytadiene appeared consistently in all five extracts with concentrations varying from 0.72% (STDE) to 3.16% (SADE). Species‐distinctive minor constituents included β‐caryophyllene (SGDE only, 0.26%), guaiol predominantly in SPDE and SATDE (0.64%–0.75%), epimanool and manool detected uniquely in STDE (0.55%–0.50%), and dehydrovomifoliol showing substantial elevation in SATDE (2.34%) relative to trace amounts in other species. Tocopherolic constituents were universally present across all species, with γ‐tocopherol concentrations ranging from 0.49% (STDE) to 1.36% (SATDE) and α‐tocopherol ranging from 0.34% (STDE) to 3.62% (SATDE), with SATDE demonstrating notably elevated tocopherol levels. Monoterpenoids represented minor yet consistent components across all five species at <0.04%, including β‐pinene, eucalyptol, and limonene. The comparative analysis demonstrates that all five *Strobilanthes* species share fundamental triterpene‐sterol and aliphatic hydrocarbon chemotypes, while exhibiting species‐specific quantitative and qualitative variations that define their individual chemical profiles and potentially contribute to their distinct ethnopharmacological properties. TIC Profiles and Structures of major components of SGDE, SPDE, SADE, SATDE, and STDE are depicted in Figures 1 and 2.

**TABLE 2:** Comparative Chemical Composition (major phytochemicals) of Extracts of SGDE, SPDE, SADE, SATDE, and STDE.

S. No.	Compound	*RI* ^Calc^	RI^DB^	SGDE	SPDE	SADE	SATDE	STDE
1.	Sclareol	515‐03‐7	2218	2.69	—	—	—	10.72
2.	*E*‐2‐decenoic acid	1425	—	—	1.00	—	—	—
3.	Dehydrovomifoliol	1790	1800 ± N/A	0.43	0.62	—	2.34	0.53
4.	Neophytadiene	1831	1838 ± 3	1.22	1.98	3.16	1.60	0.72
5.	Hexafarnesylacetone	1846	1844 ± 4	1.23	—	1.36	0.47	0.52
6.	Hexadecanoic acid	1966	1962 ± N/A	0.30	2.80	2.71	2.28	—
7.	Methyl linolenate	2100	2099 ± 3	—	—	—	3.41	1.72
8.	Linolenic acid	2102	2139 ± N/A	0.11	—	3.51	0.51	0.82
9.	Phytol	2114	2114 ± 6	8.12	1.86	3.21	2.88	5.81
10.	Linoleic acid	2141	2139 ± N/A	—	2.53	1.13	4.03	—
11.	Phytyl 2‐methylbutanoate	2434	2441 ± N/A	1.96	0.16	0.25	0.01	—
12.	2—methyltetracosane	2464	2462 ± 2	0.55	—	1.35	—	1.03
13.	n‐heptacosane	2700	2700	1.57	0.47	1.43	2.90	1.48
14.	Squalene	2829	2827 ± 8	2.19	1.01	2.23	2.57	2.51
15.	2‐methyloctacosane	2863	2863 ± 2	0.54	0.11	2.12	—	1.45
16.	n‐nonacosane	2900	2900	2.39	1.11	4.69	1.08	3.37
17.	n‐triacontane	3000	3000	0.52	0.75	1.52	1.14	0.44
18.	γ‐tocopherol	3049	3064 ± N/A	0.78	0.82	0.74	1.36	0.49
19.	2‐methyltriacontane	3063	3062 ± 2	0.89	—	0.84	3.61	1.03
20.	n‐hentriacontane	3100	3100	3.70	1.95	5.80	1.40	4.22
21.	α‐tocopherol	3130	3131 ± N/A	0.67	0.86	0.60	3.62	0.34
22.	24‐methylenecholesterol	3208	—	—	—	1.35	—	—
23.	24‐epicampesterol	3215	2632 ± N/A	—	—	2.23	7.89	2.27
24.	Stigmasterol	3246	3249 ± N/A	5.84	4.64	6.92	0.80	5.16
25.	n‐tritriacontane	3300	3300	4.81	—	—	0.01	4.49
26.	β‐sitosterol	3311	3308 ± N/A	11.28	10.10	11.29	1.48	8.17
27.	β‐amyrin	3331	3336 ± N/A	1.32	1.47	4.44	1.64	1.32
28.	β‐amirone	3356	—	—	—	0.46	5.34	0.01
29.	Lupen‐3‐one	3361	3395 ± 11	—	7.44	—	—	—
30.	Lupeol	3380	3389 ± N/A	5.85	22.63	9.07	1.34	5.74
31.	Glutin‐5‐en‐3β‐ol	3391	—	—	—	1.81	—	—
32.	γ‐sitostenone	3444	—	—	4.24	—	—	—
33.	Fridelan‐3‐one	3514	3533 ± 22	—	—	1.39	—	—
34.	Stigmastane‐3,6‐dione	3606	3601 ± N/A	0.43	—	0.34	1.47	0.43
35.	Betulin	3730	3740 ± 21	—	1.18	0.17	—	0.25

RI^Calc^, calculated retention indices; RI^DB^, Literature retention indices value on a DB‐5 MS column in reference Adams [31] and NIST web book.

### 3.2 Chemometric Analysis

**FIGURE 3:**
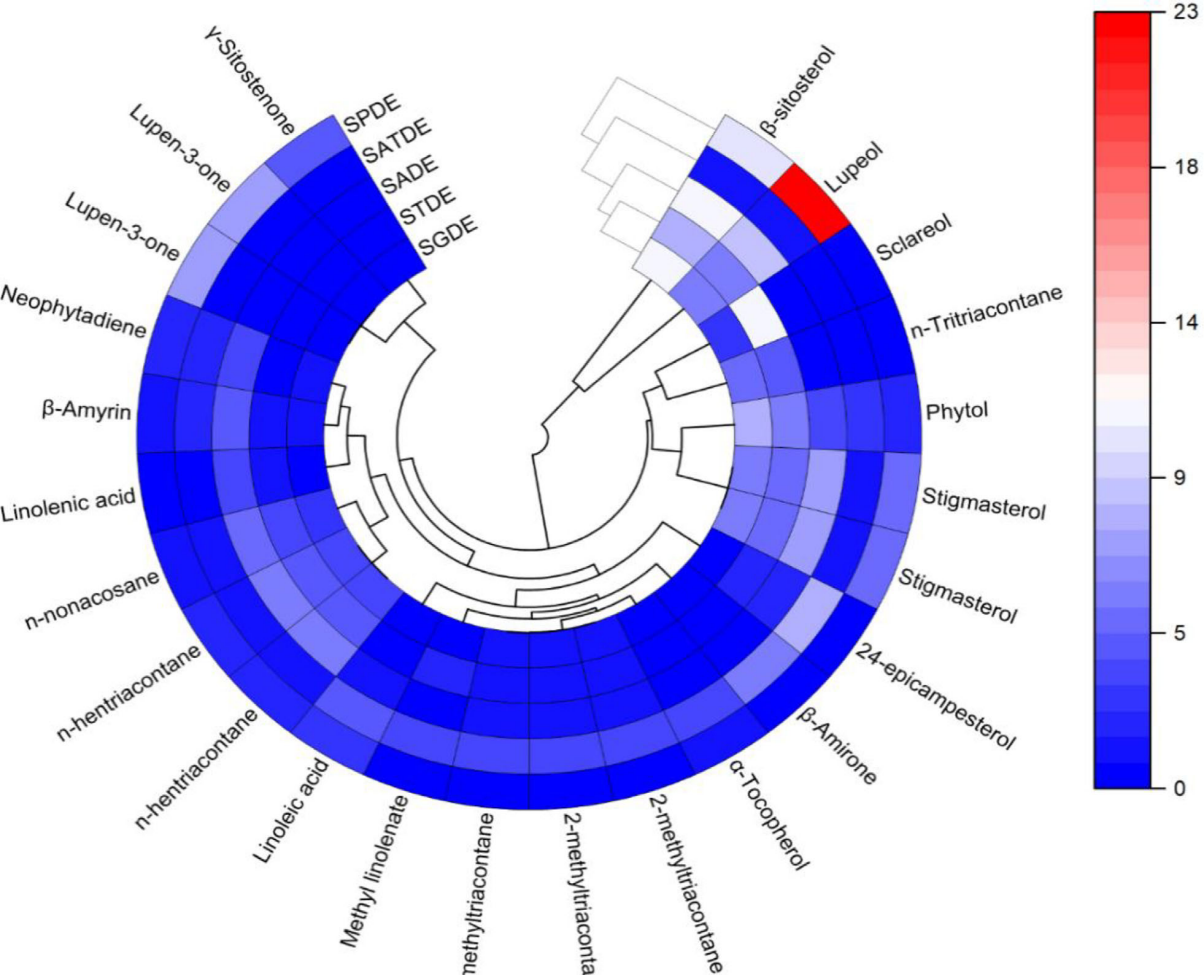
Heat Map of major bioactive compounds of five *Strobilanthes* species.

The heatmap (Figure 3) provide clear visualization and statistical interpretation of the phytochemical variation among five *Strobilanthes* species. The heatmap reveals two primary chemotypic clusters based on the relative abundance of 24 major compounds: SGDE, SPDE, and SADE characterized by high β‐sitosterol (10.10%–11.29%) and elevated lupeol (notably 22.63% in SPDE); and SATDE and STDE distinguished by higher levels of 24‐epicampesterol (7.89%), β‐amirone (5.34%) and methyl linolenate. STDE uniquely shows abundant sclareol (10.72%), absent or minimal in other species. Phytol, stigmasterol and neophytadiene are distributed across all species at moderate levels.

### 3.3 Principal Component Analysis

**FIGURE 4:**
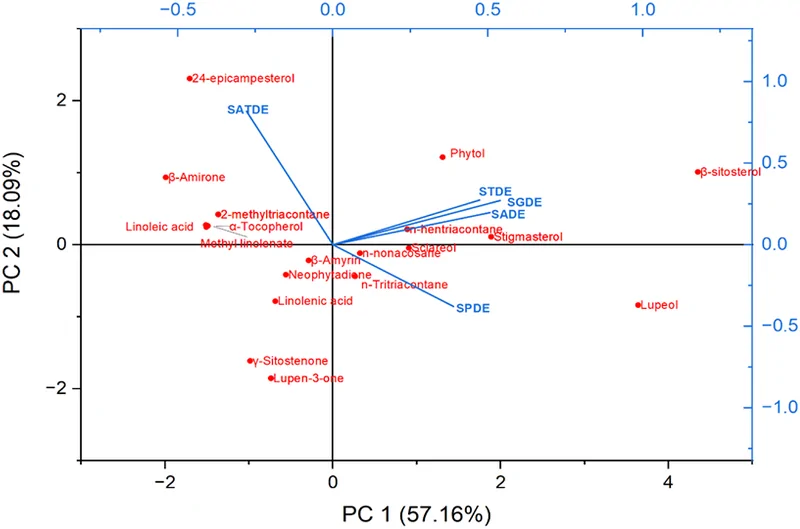
PCA of major bioactive compounds of five *Strobilanthes* species.

Principal component analysis (PCA) (Figure 4) explained 75.25% of total phytochemical variance, with PC1 (57.16%) driven by sterols and triterpenes such as β‐sitosterol, lupeol, stigmasterol, and n‐hentriacontane and PC2 (18.09%) influenced by oxygenated diterpenoids and sterol derivatives like 24‐epicampesterol, β‐amirone and phytol. Species groupings along PC1 and PC2 align with the heatmap clusters, validating chemotaxonomic differentiation, and identifying key marker compounds for species authentication and phytochemical characterization.

### 3.4 Antioxidant Activity

The antioxidant capacity of five *Strobilanthes* extracts (SGDE, SPDE, SADE, SATDE and STDE) and the reference antioxidant BHT was evaluated by the DPPH assay at concentrations of 125, 250, 500, and 1000 µg/mL. All samples exhibited a concentration‐dependent increase in DPPH radical scavenging, as shown in Table 2. Among the extracts, STDE demonstrated the strongest activity, with inhibition values rising from 25.21%  ±  0.13 at 125 µg/mL to 63.33%  ±  0.13 at 1000 µg/mL. SGDE also showed pronounced activity, increasing from 21.72%  ±  0.11 to 71.73%  ±  0.08 over the concentration range. Conversely, SPDE presented the lowest scavenging effect, ranging from 5.52%  ±  0.18 to 52.56%  ±  0.65. BHT exhibited consistently superior activity, with percent inhibition from 91.57%  ±  0.07 at the lowest concentration to 97.23%  ±  0.05 at the highest.

The IC_50_ values further emphasized these trends (Table 3). STDE achieved the lowest IC_50_ (indicating highest potency), while SADE (532.15 µg/mL) showed the highest IC_50_ value (low potency). Higher IC_50_ values observed for SGDE (465.45 µg/mL), SPDE (460.49 µg/mL), SATDE (410.25 µg/mL), and SADE (532.15 µg/mL) reflected their relatively weaker antioxidant capacities. Overall, these results identify STDE as possessing particularly potent radical scavenging activity, with an IC_50_ value nearest to BHT (standard used), whereas BHT remained the most effective across all concentrations tested.

**TABLE 3:** DPPH Radical Scavenging Activity of *Strobilanthes* extracts and BHT at various concentrations (Mean  ±  SE).

S. No.	Samples	Concentration (µg/mL)	% DPPH Radical Scavenging Activity	IC_50_ values (µg/mL)
1.	SGDE	125	21.71 ± 0.11^q^	465.44
		250	31.41 ± 0.15^no^	
		500	49.171 ± 0.180^j^	
		1000	71.72 ± 0.07^e^	
2.	SPDE	125	5.51 ± 0.17^u^	460.49
		250	14.03 ± 0.76^t^	
		500	31.85 ± 0.17^n^	
		1000	52.55 ± 0.65^i^	
3.	SADE	125	17.26 ± 0.02^r^	532.15
		250	21.83 ± 0.08^q^	
		500	34.68 ± 0.06^m^	
		1000	54.66 ± 0.12^h^	
4.	SATDE	125	15.50 ± 0.08^s^	410.25
		250	30.89 ± 0.04°	
		500	38.78 ± 0.09^l^	
		1000	56.39 ± 0.12^g^	
5.	STDE	125	25.20 ± 0.12^p^	235.73
		250	46.72 ± 0.15^k^	
		500	55.47 ± 0.12^h^	
		1000	63.32 ± 0.12^f^	
6.	BHT^*^	125	40.34 ± 0.33^i^	165.45
		250	63.06 ± 0.1.15^d^	
		500	95.26 ± 0.1.15^b^	
		1000	97.22 ± 0.05^a^	

Significance level: 0.05, *p* ≤ 0.05, *n* = 6, Data are represented by means ± SD of 3 independent experiments. Columns with the same letter did not differ significantly according to ANOVA analysis.

SGDE, *S. glutinosa;* SPDE, *S. penstemonoid;* SADE, *S. angustifrons;* SATDE, *S. attenuate;* STDE, *S. tomentosa;* BHT, Butylated Hydroxy Toluene.

* = Positive control.

The metal chelating abilities of five *Strobilanthes* extracts (SGDE, SPDE, SADE, SATDE, and STDE) were evaluated at concentrations of 125, 250, 500, and 1000 µg/mL, using EDTA as a standard. All extracts showed a concentration‐dependent increase in chelating activity, with results summarized in Table 4. At 1000 µg/mL, SADE (74.94  ±  1.28%) and SGDE (74.65  ±  0.87%) exhibited the highest chelation, closely followed by SATDE (65.99  ±  0.55%), while SPDE displayed the lowest activity, rising from 3.63 ± 0.23% to 31.62 ± 0.02%. EDTA achieved the strongest chelation overall, with 82.75 ± 0.93% at the highest concentration.

**TABLE 4:** Metal Chelating Activity (Mean ± SE) of *Strobilanthes* extracts and EDTA at various concentrations.

S. No.	Samples	Concentration (µg/mL)	% Metal chelating activity	IC_50_ values (µg/mL)
1.	SGDE	125	14.73 ± 0.82^mn^	435.61
		250	24.80 ± 1.44^jk^	
		500	51.59 ± 1.59^e^	
		1000	74.64 ± 0.87^b^	
2.	SPDE	125	3.62 ± 0.23°	343.94
		250	11.69 ± 0.24^n^	
		500	27.47 ± 0.19^j^	
		1000	31.62 ± 0.02^i^	
3.	SADE	125	19.65 ± 0.27^l^	400.05
		250	35.37 ± 0.69^h^	
		500	55.23 ± 0.58^d^	
		1000	74.93 ± 1.27^b^	
4.	SATDE	125	22.75 ± 0.43^kl^	307.14
		250	41.30 ± 0.24^g^	
		500	54.74 ± 0.09^de^	
		1000	65.99 ± 0.55^c^	
5.	STDE	125	15.50 ± 0.08^m^	512.94
		250	25.20 ± 0.12^jk^	
		500	38.78 ± 0.09^gh^	
		1000	63.32 ± 0.12^c^	
6.	EDTA^*^	125	27.47 ± 0.19^j^	376.34
		250	46.72 ± 0.15^f^	
		500	63.32 ± 0.12^c^	
		1000	82.75 ± 0.92^a^	

Significance level: 0.05, p ≤ 0.05, n = 6, Data are represented by means ± SD of 3 independent experiments. Columns with the same letter did not differ significantly according to ANOVA analysis.

SGDE, *S. glutinosa;* SPDE, *S. penstemonoid;* SADE, *S. angustifrons;* SATDE, *S. attenuate;* SATDE, *S. tomentosa;* EDTA, Ethylene Diamine Tetra Acetic Acid.

* = Positive control.

IC_50_ values (Table 4) revealed that SATDE (307.15 µg/mL) was the most potent among the extracts, with EDTA showing the highest efficacy at 376.35 µg/mL. Other IC_50_s included SPDE (343.94 µg/mL), SADE (400.05 µg/mL), SGDE (435.62 µg/mL) and STDE (512.94 µg/mL).

### 3.5 Antibacterial Activity

**FIGURE 5:**
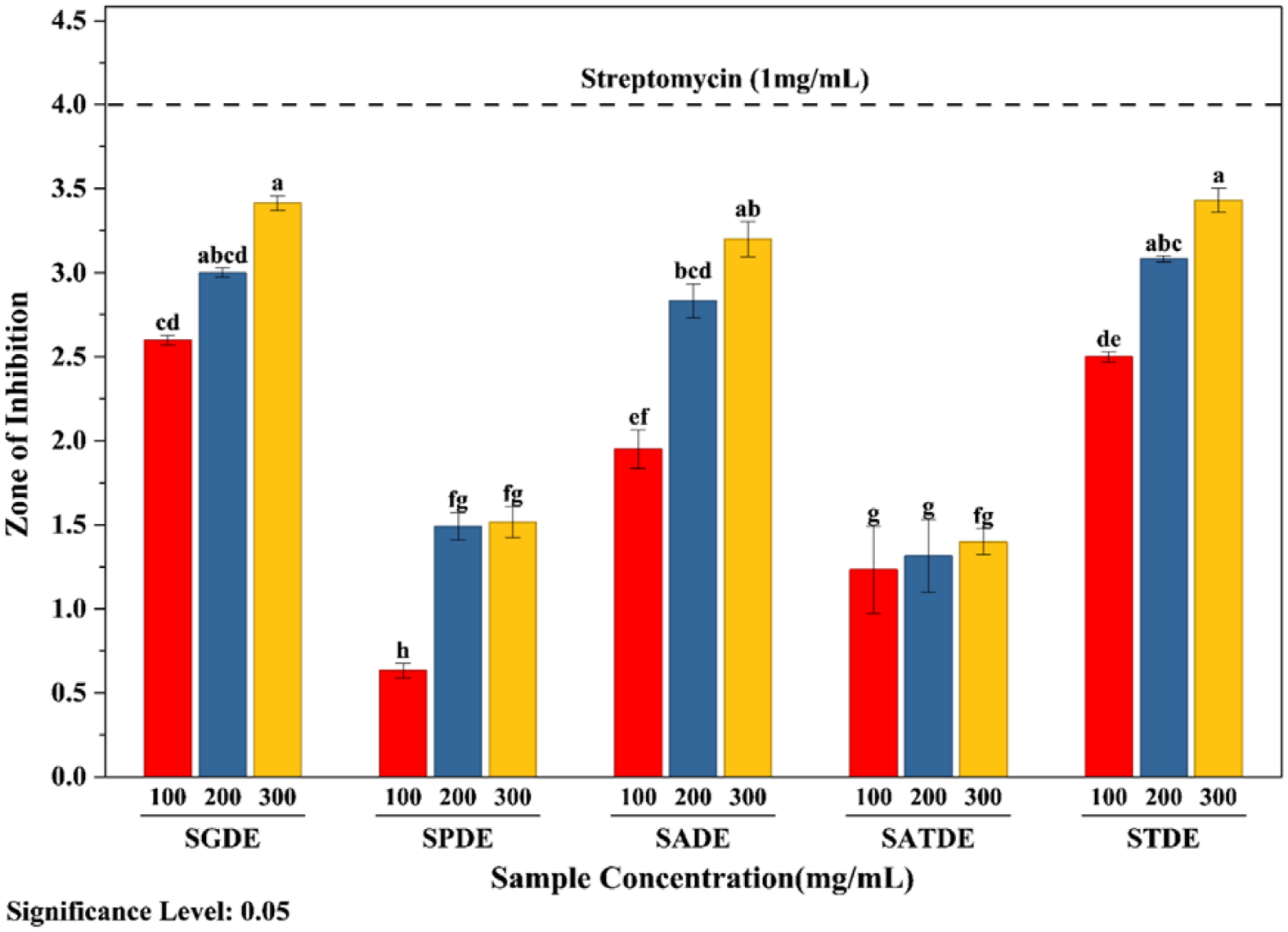
Comparative Antibacterial Activity of SGDE, SPDE, SADE, SATDE, and STDE extracts against *A. salmonicida*: Zone of Inhibition Assay compared to Streptomycin. *p* ≤ 0.05, *n* = 6, Data are represented by means ± SD of 3 independent experiments. Columns with the same letter did not differ significantly according to ANOVA analysis.

The antibacterial activity of five *Strobilanthes* extracts (SGDE, SPDE, SADE, SATDE, and STDE) against *Aeromonas salmonicida* was evaluated at 100, 200 and 300 mg/mL concentrations as shown in Figure 5. SGDE and STDE exhibited the strongest antibacterial effects, with inhibition increasing significantly from approximately a net zone of inhibition of 2.6‐2.5 mm at 100 mg/mL to around 3.4 mm at 300 mg/mL. SADE also showed notable activity, achieving an inhibition of 3.2 at the highest concentration. In contrast, SPDE and SATDE displayed minimal activity, with inhibition remaining below 1.52 across all concentrations. The positive control group (streptomycin) showed the highest inhibition (4.03 mm  ± 0.088) consistently at 1 mg/mL. These results demonstrate a clear concentration‐dependent antibacterial response, identifying SGDE and STDE as the most effective extracts against *A. salmonicida*.

**FIGURE 6:**
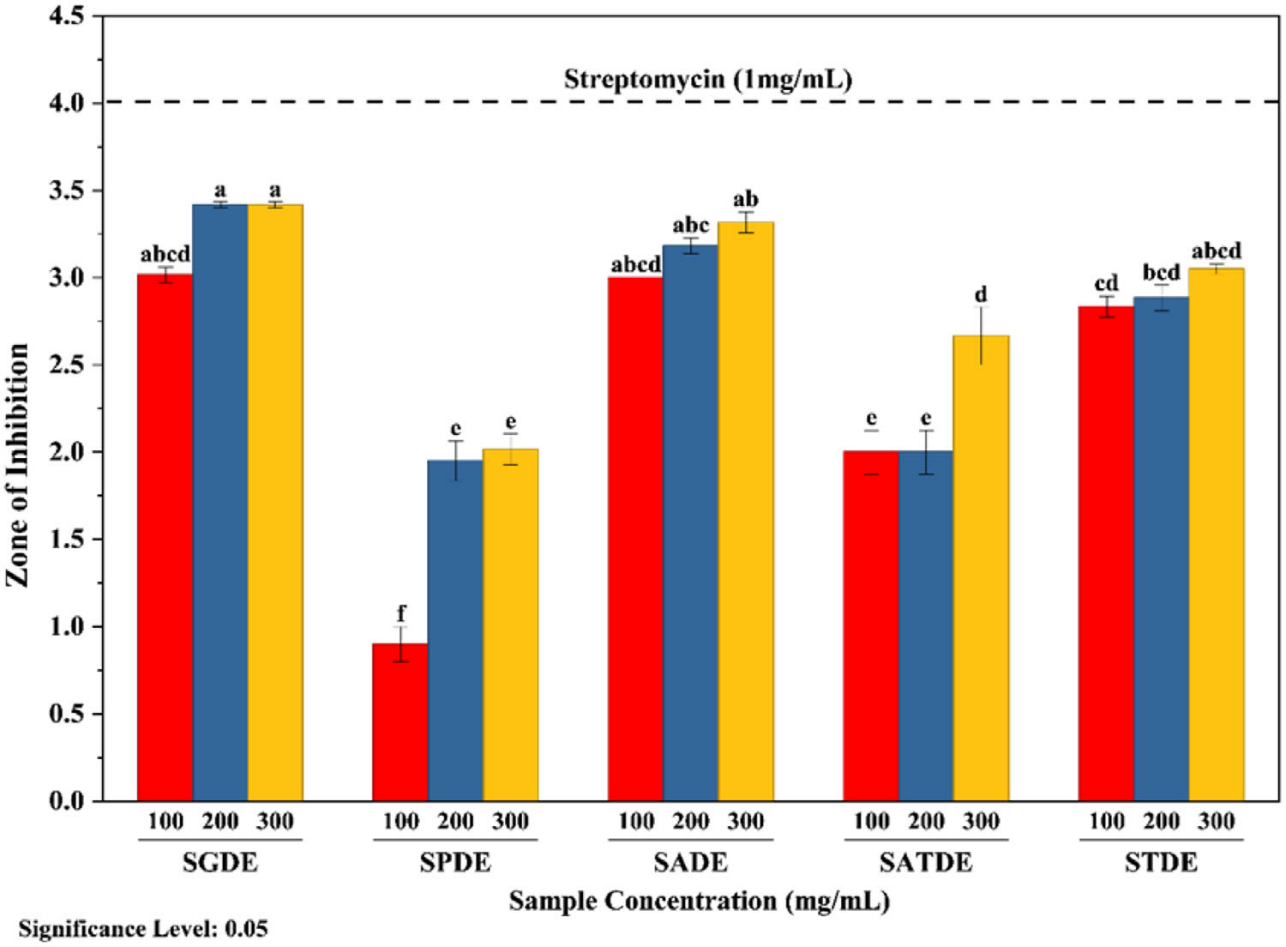
Comparative Antibacterial Activity of SGDE, SPDE, SADE, SATDE, and STDE Extracts against *E. tarda*: Zone of Inhibition Assay Compared to Streptomycin. *p* ≤ 0.05 *n* = 6, Data are represented by means ± SD of 3 independent experiments. Columns with the same letter did not differ significantly according to ANOVA analysis.

The antimicrobial activity of extracts from five *Strobilanthes* species against *Edwardsiella tarda* was systematically assessed at concentrations of 100, 200, and 300 mg/mL, with results summarized in Figure 6. The findings revealed a dose‐dependent increase in antibacterial effects for the most active extracts. SGDE and STDE exhibited the highest inhibitory effects, with mean inhibition values rising from approximately 1.95–2.5 at 100 mg/mL to around 3.2–3.43 at 300 mg/mL. SADE demonstrated moderate activity, whereas SPDE and SATDE showed minimal effects across all concentrations. The positive control group had the highest inhibition, with a net zone of inhibition, with a mean of 4.03 ± 0.088 at 1 mg/mL. These results highlight the significant antibacterial potential of SGDE and STDE against *E. tarda*, supporting their possible application in aquaculture health management and antimicrobial development. Dose‐dependent inhibition zones of SGDE and STDE extracts and SADE are depicted in Figure 7a–c.

FIGURE 7(a) Negative control and streptomycin (positive control) plate showing inhibition zones in the antimicrobial assay. (b) Dose‐dependent inhibition zones of SGDE and STDE extracts against *A. salmonicida* observed by disc diffusion assay. (c). Dose‐dependent inhibition zones of SGDE and SADE extracts against *E. tarda* observed by disc diffusion assay.

**Figure:**
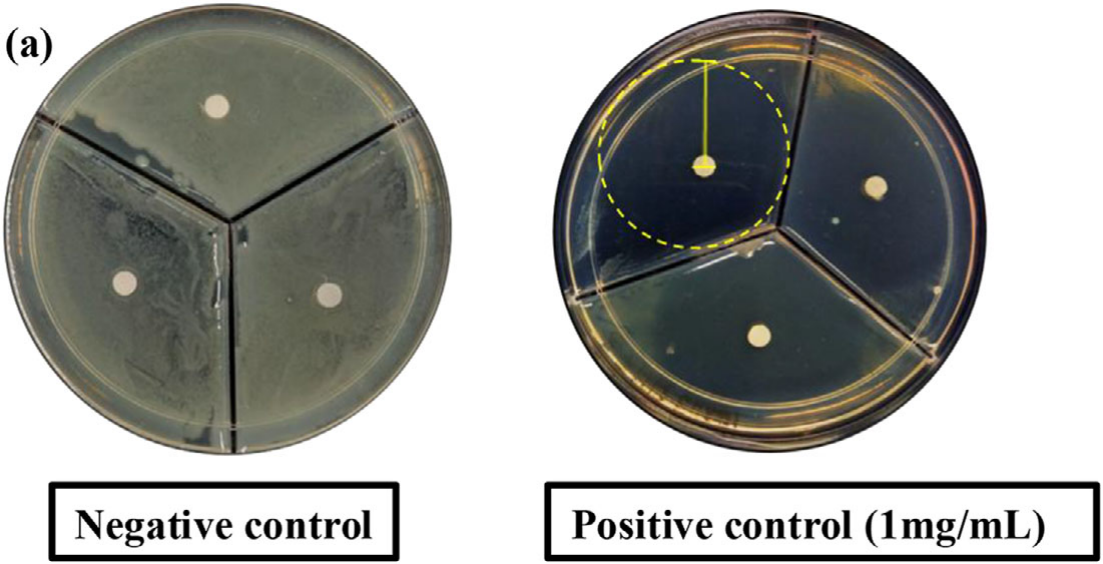

**Figure:**
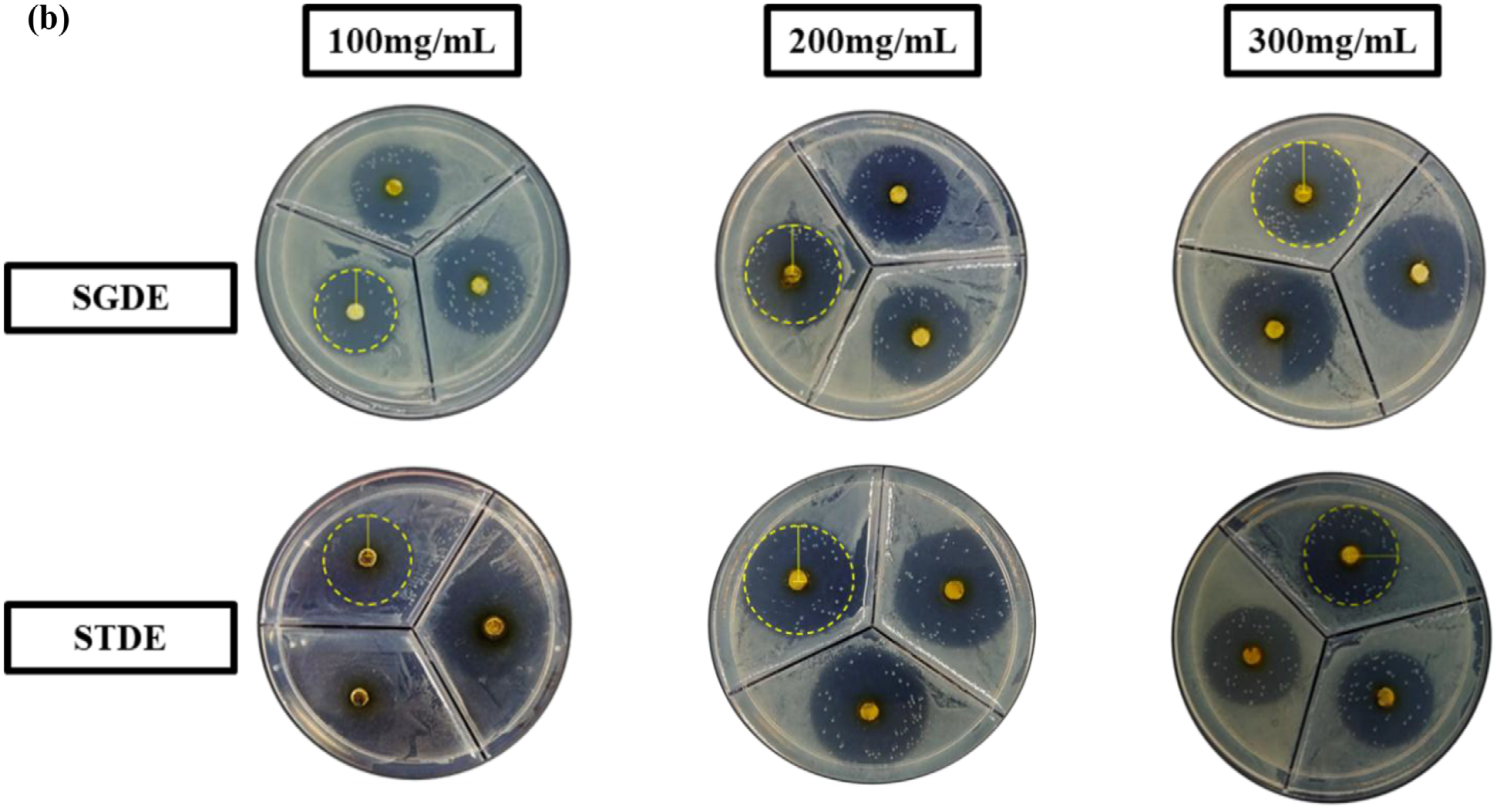

**Figure:**
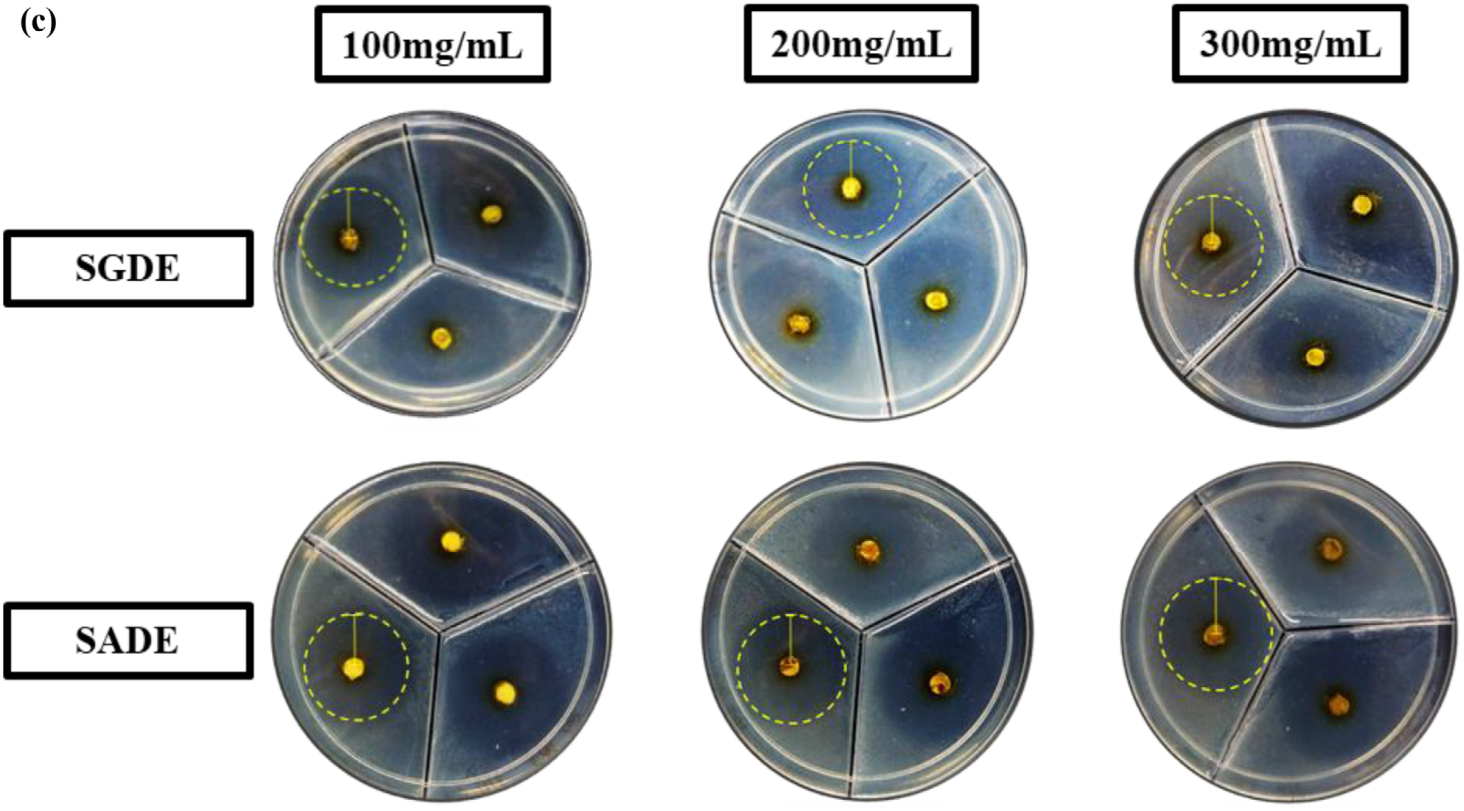

These findings align with earlier studies reporting strong antibacterial activities of other *Strobilanthes* species, such as *S. glutinosus* against *Staphylococcus aureus*, *S. crispus* methanolic extract against *Bacillus cereus*, *S. heyneanus* root extract's broad‐spectrum efficacy and crude leaf extract active against multiple pathogens comparable to standard antibiotics [68, 69, 70]. Additionally, *S. auriculata's* antibacterial effects against *Salmonella typhimurium* have been linked to its high phenolic content and antioxidant capacity [71].

The superior antibacterial performance of SGDE and STDE is attributed to high concentrations of bioactive compounds with known antimicrobial attributes, primarily β‐sitosterol and stigmasterol (11.3% and 5.8% in SGDE; 8.2% and 5.2% in STDE), which disrupt bacterial cell wall synthesis despite limited direct antibacterial action [72, 73]. The presence of phytol (8.1% in SGDE; 5.8% in STDE), a diterpene with strong bactericidal activity, particularly against *Pseudomonas aeruginosa*, further supports the observed effects [74]. Linoleic and linolenic acids, also detected, contribute antibacterial properties with low minimum inhibitory concentrations against pathogens like *B. cereus* and *S. aureus* [75, 76].

The concentration‐dependent antibacterial responses suggest synergistic interactions among terpenoid antioxidants, sterols and fatty acids, providing a multi‐targeted antimicrobial mechanism. The efficacy against two important aquaculture pathogens, *A. salmonicida* and *E. tarda*, highlights these *Strobilanthes* extracts' potential for aquaculture disease management and substantiates their traditional use in treating bacterial infections. Further research into their minimum inhibitory concentrations and molecular mechanisms is warranted.

### 3.6 Phytochemical‐Bioactivity Correlation Analysis

**FIGURE 8:**
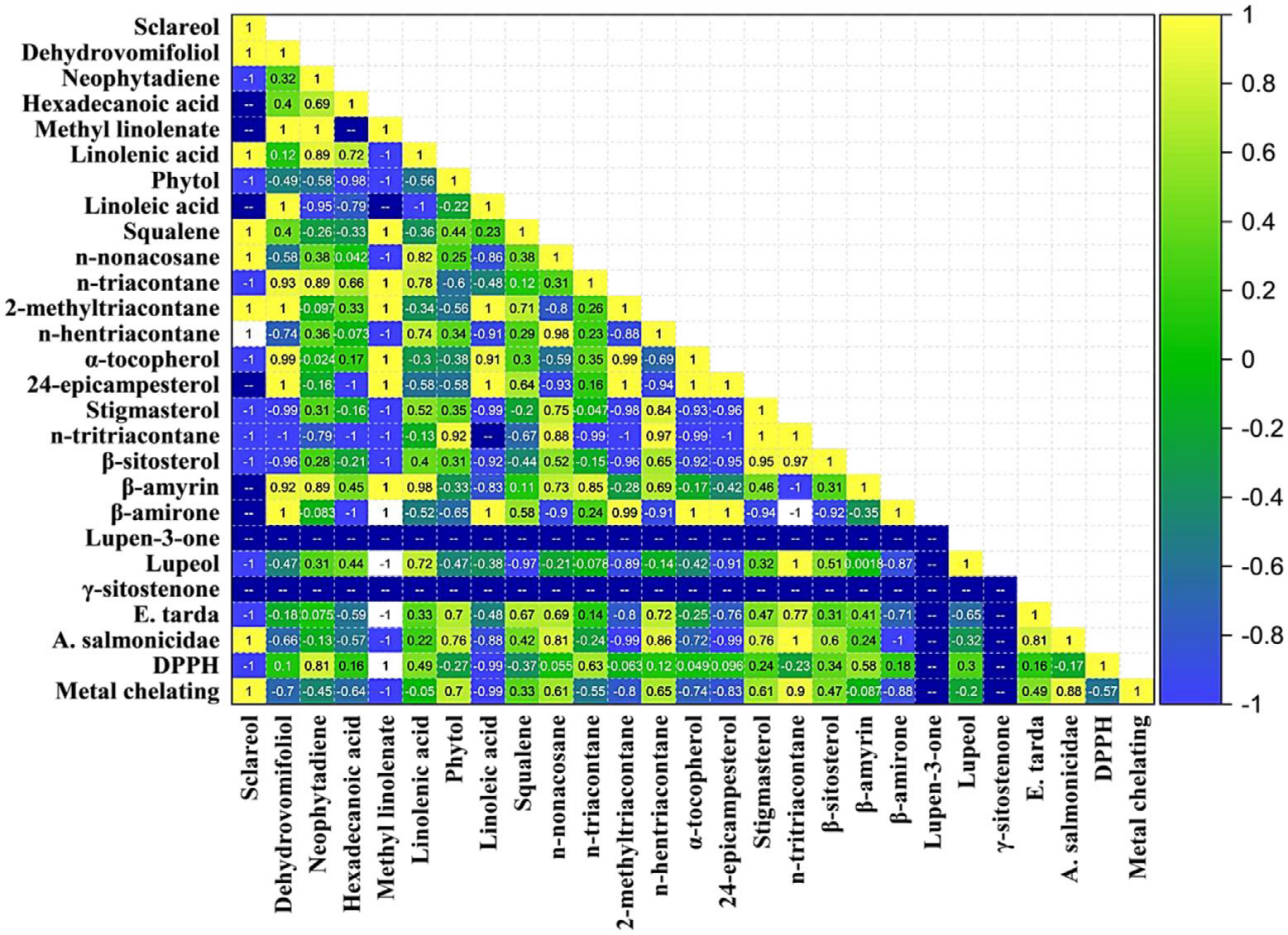
Correlation Matrix of Phytochemical compounds with antioxidant and antibacterial activities.

**FIGURE 9:**
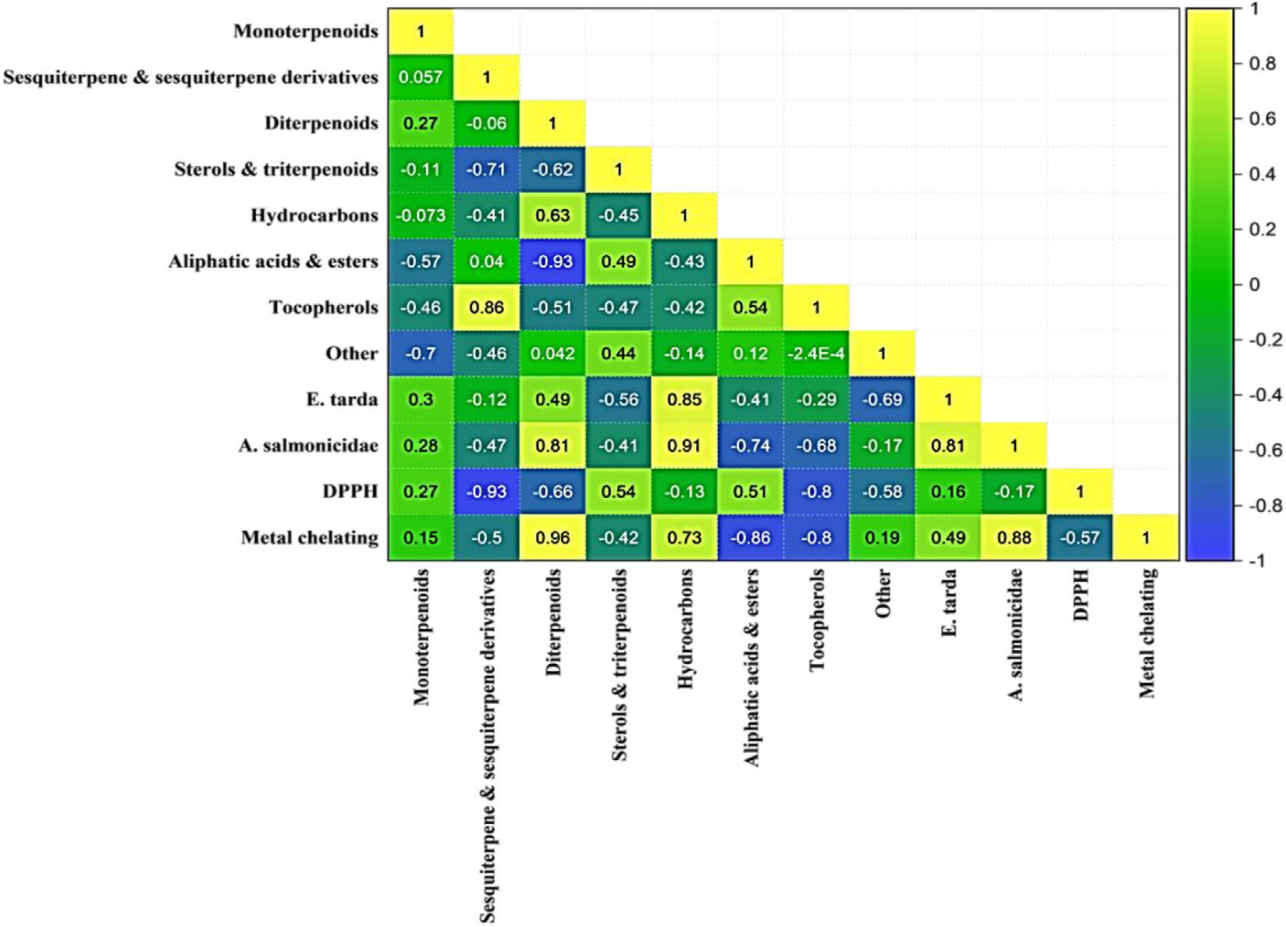
Correlation Matrix of Phytochemical Class with antioxidant and antibacterial activities.

Pearson correlation analysis identified significant associations between specific phytochemical constituents and measured bioactivities. At the individual compound level (Figure 8), n‐triacontane showed moderate positive correlation with DPPH radical scavenging (*r* = 0.63). At the same time, n‐tritriacontane exhibited strong positive correlation with metal chelating activity (*r* = 0.90), which means they are associated with antioxidant compounds. Notably, sclareol demonstrated perfect correlation with *A. salmonicida* inhibition (*r* = 1.00), which may be due to its high percentage abundance and was also confirmed by its molecular docking score (significant binding affinity: −6.4 and 5.3 kcal/mol). and n‐tritriacontane with *E. tarda* inhibition (*r* = 1.00), suggesting these hydrocarbons are associated with antimicrobial phytocompounds for antimicrobial efficacy. Additional compounds showing intermediate positive correlations with both bacterial pathogens included stigmasterol, n‐nonacosane, squalene, and phytol (*r* = 0.47‐0.68), indicating synergistic contribution of sterols and diterpenoids to antimicrobial effects. At the phytochemical class level (Figure 9), diterpenoids exhibited strong positive correlation with metal chelating activity (*r* = 0.96), whereas sesquiterpenes showed strong negative correlation with DPPH scavenging (*r* = −0.93). For antibacterial effects, diterpenoids and hydrocarbons correlated positively with *A. salmonicida* inhibition (*r* = 0.81 and 0.63, respectively), contrasting sharply with sterols and triterpenoids, which demonstrated strong negative correlations with both *E. tarda* (*r* = −0.56) and *A. salmonicida* (*r* = −0.41) inhibition. These findings suggest that while sterols and triterpenoids contribute to the extract's general antioxidant framework and bioavailability, diterpenoids and long‐chain hydrocarbons represent the associations with active constituents for antimicrobial activity against tested aquaculture pathogens [6]. The correlation coefficients were calculated using all available experimental data points. The occurrence of *r* = 1.00 likely reflects the limited sample size and strong linear trends within the dataset; therefore, these results should be interpreted with caution and validated in future studies using larger datasets.

### 3.7 Molecular Docking Analysis

Molecular docking was performed to validate antibacterial potential of major phytocompounds (high percentage) by elucidating the binding interactions of major phytocompounds from five *Strobilanthes* species with protein targets 4DR3 and 1CY8. Three‐dimensional and two‐dimensional structural visualizations (Figure 10a,b) illustrated ligand orientations within protein binding sites and identified key amino acid residues mediating interactions. The docking grid parameters were automatically defined by CB‐Dock2 according to the geometry of the predicted binding pockets. The selected cavities exhibited volumes of 6756 Å^3^ for 4DR3 and 2128 Å^3^ for 1CY8, with corresponding grid center coordinates of (*X* = 91, *Y* = 77, *Z* = 20) and (*X* = 52, *Y* = 22, *Z* = 45), respectively. The predominant interaction types included van der Waals and alkyl contacts, with occasional unfavorable donor–donor interactions, indicating stable and diverse binding profiles.

FIGURE 10(a) 2D and 3D ligand‐protein interaction analysis for protein 4DR3. (b) 2D and 3D ligand‐protein interaction analysis for protein 1CY8.

**Figure:**
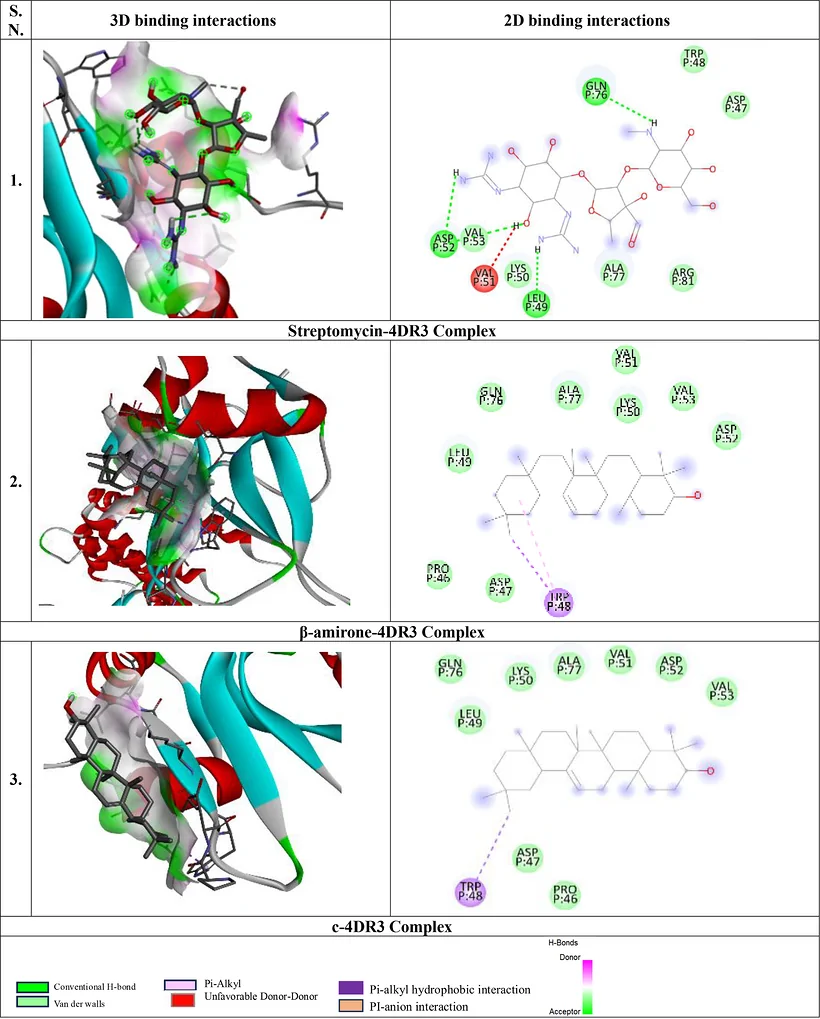

**Figure:**
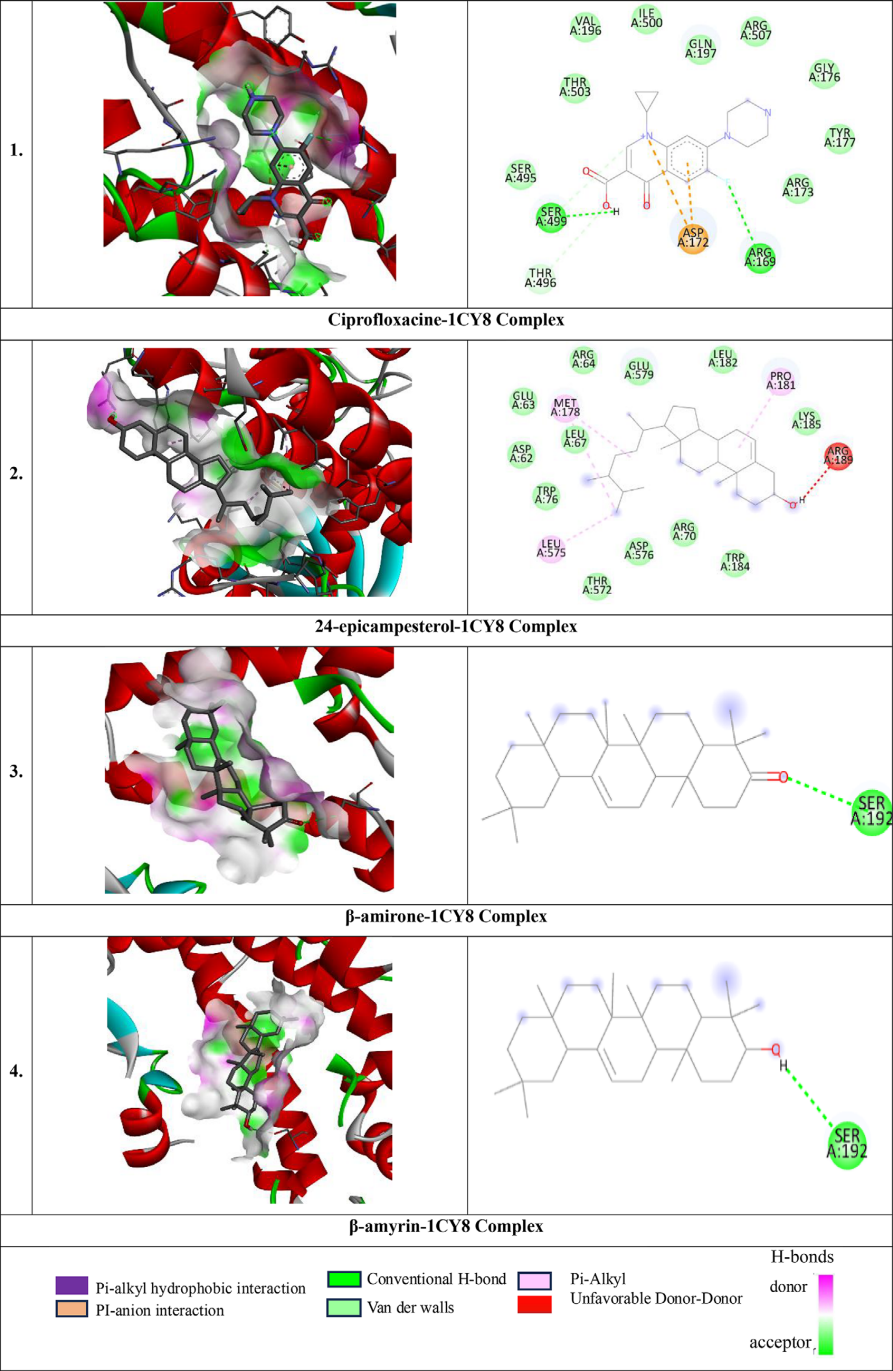

Binding affinity data (Table 5) revealed that β‐amyrin (Vina scores: −7.3 kcal/mol for 4DR3 and −7.6 kcal/mol for 1CY8), β‐amirone (−7.0 kcal/mol for 4DR3 and −8.2 kcal/mol for 1CY8) and 24‐epicampesterol (‐6.6 kcal/mol for 4DR3 and ‐7.6 kcal/mol for 1CY8) exhibited superior affinities compared to reference standards. In the β‐amirone‐4DR3 complex, van der Waals interactions predominated with residues LEU‐49, PRO‐46, GLN‐76, and VAL‐31 accompanied by a Pi‐Sigma aromatic interaction with TRP‐48. The β‐amirone‐1CY8 interaction was limited to a single van der Waals contact with SER‐192. β‐Amyrin formed extensive van der Waals interactions with GLN‐76, LYS‐50, and PRO‐46 at 4DR3, with an accompanying Pi‐Sigma interaction at TRP‐48, while 1CY8 binding involved only a van der Waals contact with SER‐192. For 24‐epicampesterol, the 4DR3 complex displayed van der Waals interactions at ALA‐47, LEU‐49, and VAL‐53, whereas the 1CY8 complex showed a more diverse binding pattern including multiple van der Waals contacts (GLU‐63, ARG‐64, TRP‐76), a hydrogen bond with ARG‐189 and alkyl interactions involving MET‐178, PRO‐181, and LYS‐185.

**TABLE 5:** Binding affinity of major *Strobilanthes* phytocompounds against 4dr3 and 1cy8 protein targets.

S. No.	Compound	CID No.	Vina score (4dr3) (kcal/mol)	Vina score (1cy8) (kcal/mol)
1.	Lupeol	259846	−6.4	−7.3
2.	β‐sitosterol	222284	−6.5	−7.5
3.	Sclareol	163263	−5.3	−6.4
4.	Phytol	5280435	−4.5	−5.4
5.	Stigmasterol	5280794	−6.5	−7.4
6.	24‐epicampesterol	5283637	−6.6	−7.6
7.	Lupen‐3‐one	92158	−6.7	−7.5
8.	β‐amirone	60139487	−7.0	−8.2
9.	β‐amyrin	73145	−7.3	−7.6
10.	Epi‐lupeol	5270628	−6.4	−7.3
11.	γ‐sitostenone	457801	−6.2	−7.2
12.	α‐tocopherol	14985	−4.9	−6.7

These findings indicate that van der Waals forces constitute the primary stabilizing force across both protein targets, while aromatic, hydrogen bonding, and alkyl interactions contribute selectively to binding affinity and specificity. β‐Amyrin and β‐amirone emerge as promising phytoconstituents, warranting further pharmacological investigation and drug development research. Collectively, these findings indicate that most major compounds exhibited high binding affinity toward the target protein, supporting their potential antimicrobial activity. Correlation analysis further suggests that hydrocarbons such as n‐tritriacontane, along with diterpenoids, triterpenoids, and phytosterols may contribute synergistically to the observed antimicrobial effects.

### 3.8 ADMET and Pharmacokinetic Assessment

Comprehensive in silico ADMET profiling of 15 major phytochemicals from *Strobilanthes* species (Table 6): lupeol (1), β‐sitosterol (2), sclareol (3), phytol (4), stigmasterol (5), 24‐epicampesterol (6), lupen‐3‐one (7), n‐hentriacontane (8), β‐amirone (9), n‐tritriacontane (10), n‐nonacosane (11), β‐amyrin (12), epi‐lupeol (13), γ‐sitostenone (14), and α‐tocopherol (15) was conducted to evaluate their pharmaceutical potential. All compounds satisfied Lipinski's rule of five (molecular weight 296‐465 Da). Compounds 3, 5 and 6 demonstrated superior drug‐likeness (QED 0.46‐0.74) with moderate lipophilicity (4.70‐7.80), while compounds 8, 10, 11, and 15 exhibited substantially compromised profiles with excessive lipophilicity (LogP >11) and poor aqueous solubility (logS <−7.8). Absorption and distribution analysis demonstrated high intestinal absorption (≥0.99) and favorable bioavailability (0.76‐0.81) for compounds 3, 5, 9, 12, 14, and 15, except compounds 8 and 10 with reduced bioavailability (0.38–0.40). Blood‐brain barrier penetration was significantly elevated in compounds 3, 4, 9, 11, 12, and 14 (≥0.85). Plasma protein binding was exceptionally high (≥82.66%), indicating substantial systemic persistence. Metabolic assessment revealed minimal cytochrome P450 inhibition for most compounds, though compounds 3, 9, 11, and 15 demonstrated elevated CYP2C9/2D6 substrate probabilities. Half‐lives ranged from 0.00–158.41 h, with compounds 4, 8, 10 and 11 exceeding 100 h, raising accumulation concerns. Toxicological profiling identified moderate‐to‐high hERG channel inhibition (0.55‐0.92), particularly in compounds 4 and 15 (0.90‐0.92), indicating cardiotoxic risk. Clinical toxicity remained uniformly low (<0.09). Carcinogenicity was substantially elevated in compounds 4, 8, 10, and 11 (0.57‐0.62), while acute toxicity was elevated in compounds 9, 10, 11, and 15.

**TABLE 6:** Predicted ADMET profiles of the top 15 major phytochemicals from *Strobilanthes* species.

Property	1	2	3	4	5	6	7	8	9	10	11	12	13	14	15
Molecular weight (Dalton)	426.73	414.72	308.51	296.54	412.70	400.69	424.71	436.85	424.71	464.91	408.80	426.73	426.73	412.70	430.72
LogP	8.02	8.02	4.70	6.36	7.80	7.63	8.23	12.34	8.38	13.12	11.56	8.17	8.02	8.23	8.84
Hydrogen bond acceptors	1.00	1.00	2.00	1.00	1.00	1.00	1.00	0.00	1.00	0.00	0.00	1.00	1.00	1.00	2.00
Hydrogen bond donors	1.00	1.00	2.00	1.00	1.00	1.00	0.00	0.00	0.00	0.00	0.00	1.00	1.00	0.00	1.00
Lipinski rule of 5	3.00	3.00	4.00	3.00	3.00	3.00	3.00	3.00	3.00	3.00	3.00	3.00	3.00	3.00	3.00
Quantitative estimate of druglikeness (QED)	0.42	0.44	0.74	0.39	0.46	0.47	0.39	0.11	0.36	0.09	0.12	0.39	0.42	0.43	0.36
Topological polar surface area (TPSA) (Å)^2^	20.23	20.23	40.46	20.23	20.23	20.23	17.07	0.00	17.07	0.00	0.00	20.23	20.23	17.07	29.46
Human intestinal absorption	1.00	1.00	1.00	1.00	1.00	1.00	1.00	1.00	1.00	1.00	1.00	1.00	0.99	1.00	1.00
Oral bioavailability	0.53	0.78	0.81	0.70	0.79	0.76	0.67	0.40	0.83	0.38	0.42	0.73	0.51	0.79	0.78
Aqueous solubility (log(mol/L)	−7.26	−6.63	−4.99	−5.33	−6.74	−6.63	−6.63	−7.82	−6.17	−7.89	−7.76	−6.94	−7.27	−6.27	−7.46
Lipophilicity log‐ratio	4.65	4.65	3.26	4.13	4.75	4.68	4.92	5.01	5.17	5.02	5.01	5.01	4.64	5.03	4.95
P‐glycoprotein Inhibition	0.77	0.94	0.25	0.88	0.93	0.92	0.78	0.98	0.87	0.99	0.98	0.87	0.75	0.95	0.93
Blood‐Brain Barrier Penetration	0.37	0.86	0.91	0.90	0.79	0.78	0.60	0.87	0.97	0.85	0.90	0.90	0.32	0.96	0.84
CYP1A2 Inhibition	0.01	0.01	0.02	0.25	0.01	0.01	0.01	0.14	5.52e‐04	0.12	0.17	1.50e‐03	0.01	3.21e‐03	0.05
CYP2C19 Inhibition	0.02	0.03	0.18	0.03	0.09	0.05	0.03	0.02	0.06	0.02	0.02	0.03	0.02	0.14	0.01
CYP2C9 Inhibition	0.04	0.02	0.06	0.02	0.04	0.02	0.06	3.88e‐03	0.02	3.58e‐03	4.28e‐03	0.01	0.04	0.04	0.02
CYP2D6 Inhibition	0.01	0.14	0.14	0.27	0.05	0.08	0.01	0.02	0.02	0.01	0.02	0.02	0.01	0.19	0.09
CYP3A4 Inhibition	0.04	0.07	0.33	0.06	0.12	0.07	0.14	0.01	0.11	0.01	0.01	0.03	0.04	0.10	0.07
Mutagenicity	0.02	0.08	0.05	0.02	0.08	0.08	0.01	1.74e‐03	0.01	1.74e‐03	1.70e‐03	0.04	0.02	0.03	0.05
Drug‐Induced Liver Injury	0.07	0.05	0.03	0.02	0.08	0.06	0.11	0.22	0.12	0.22	0.22	0.08	0.07	0.12	0.09
Carcinogenicity	0.05	0.07	0.12	0.62	0.08	0.08	0.05	0.59	0.21	0.57	0.61	0.12	0.05	0.18	0.46
Acute toxicity LD_50_ log(1/(mol/kg))	2.67	2.63	2.25	1.53	2.89	2.53	2.85	1.26	3.04	1.27	1.24	2.90	2.68	2.38	1.93

**FIGURE 11:**
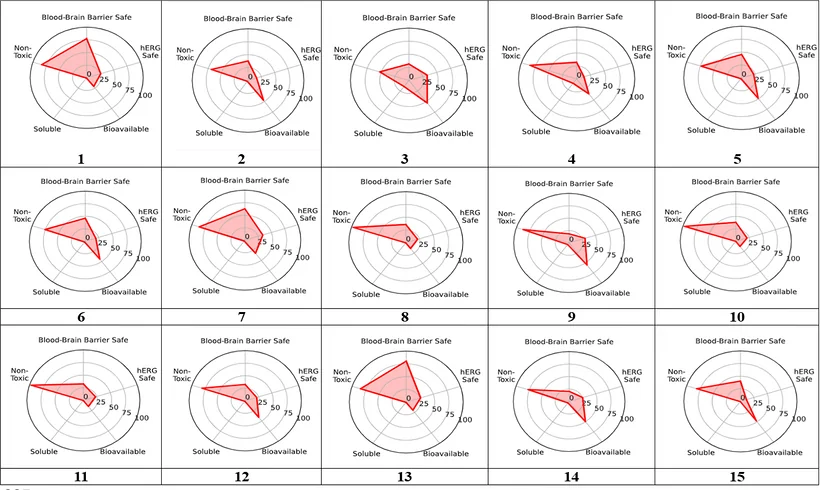
Bioavailability Radar Profiles for selected *Strobilanthes* Phytochemicals. **1**. Lupeol, **2**. β‐sitosterol, **3**. Sclareol, **4**. Phytol, **5**. Stigmasterol, **6**. 24‐epicampesterol, **7**. Lupen‐3‐one, **8**. n‐hentriacontane, **9**. β‐amirone **10**. n‐tritriacontane, **11**. n‐nonacosane, **12**. β‐amyrin, **13**. Epi‐lupeol, **14**. γ‐sitostenone, and **15**. α‐tocopherol.

Compounds 3 (sclareol), 5 (stigmasterol), and 6 (24‐epicampesterol) emerged as promising candidates owing to their favorable drug‐likeness, high bioavailability, and metabolic stability. However, toxicity prediction indicated potential cardiotoxicity, with sclareol exhibiting moderate toxicity risk, whereas stigmasterol and 24‐epicampesterol showed comparatively significant toxicity risks. Compounds 8, 10, 11 and 15 exhibited substantial liabilities requiring structural optimization. The collective lipophilicity profile (mean LogP ∼8.6) with favorable membrane permeability indicates agronomic biopesticide potential; however, environmental persistence warrants ecological assessment. These findings prioritize compounds 3, 5 and 6 for experimental validation and therapeutic development, while rational structural modifications are recommended for high‐liability compounds to optimize their ethnopharmacological utility. The Figure 11 presents bioavailability radar plots, which visually map six critical physicochemical parameters: lipophilicity, molecular size, polarity, solubility, flexibility and saturation—against optimal ranges predictive of good oral bioavailability. The radar plot displays these parameters as a red polygon within a pink shaded area that defines the ideal physicochemical space for oral absorption, systemic exposure and safety. Compounds with radar polygons fully enclosed in this optimal zone are expected to have favorable bioavailability profiles, including efficient antimicrobial activity with rapid clearance, minimal bioaccumulation and a low likelihood of off‐target effects such as blood‐brain barrier penetration. The table highlights compound 7 and 13 as particularly promising eco‐safe antimicrobial candidates due to their radar profiles predominantly within the acceptable limits, aligning with desirable efficacy and environmental safety criteria. This analysis supports early‐stage prioritization in drug development to balance therapeutic potential with ecological risk reduction.

ADMET analysis further strengthened the biological relevance of these major compounds. Sclareol, stigmasterol, and 24‐epicampesterol exhibited good binding affinity with the target protein or antimicrobial potential, superior drug‐likeness (QED 0.46–0.74), high intestinal absorption (≥0.99), favorable oral bioavailability (0.76–0.81), but sclareol showed moderate toxicity, whereas stigmasterol and 24‐epicampesterol show significant toxicity risk. In contrast, compounds such as n‐hentriacontane, n‐tritriacontane and n‐nonacosane, despite showing positive correlations with antibacterial activity, exhibited lipophilicity, and poor solubility, which may limit their pharmaceutical applicability.

## 4 Conclusion

The comprehensive phytochemical profiling and bioactivity assessment of five *Strobilanthes* species establish distinctive chemotypic signatures with significant antimicrobial potential for sustainable aquaculture disease management. SGDE and STDE emerged as efficacious extracts against the critical aquaculture pathogens *Aeromonas salmonicida* and *Edwardsiella tarda*, with enhanced antibacterial responses attributable to elevated concentrations of established antimicrobial phytoconstituents, including phytol, β‐sitosterol, and sclareol. Species‐dependent variations in antioxidant capacity were observed, with STDE demonstrating superior DPPH radical scavenging (IC_50_ = 235.73 µg/mL) and SATDE exhibiting optimal metal chelating potential (IC_50_ = 307.15 µg/mL), indicating multifaceted oxidative stress suppression mechanisms. Molecular docking and ADMET analyses identified diterpenoids and long‐chain hydrocarbons as primary antimicrobial pharmacophores, with sclareol, stigmasterol, and 24‐epicampesterol prioritized as lead candidates demonstrating favorable bioavailability but showing some toxicity risk. This research substantiates the traditional ethnopharmacological applications of *Strobilanthes* species while providing rigorous quantitative and mechanistic evidence supporting their integration as sustainable, eco‐safe alternatives to synthetic antimicrobials in aquaculture therapeutics. These findings address urgent global challenges of antibiotic resistance and environmental contamination while advancing the translational development of plant‐derived therapeutics for responsible disease management in food‐producing aquaculture systems.

## Author Contributions

**Sakshi Negi**: writing – original draft, methodology, investigation, formal analysis, data curation, formal analysis, conceptualization. **Ravendra Kumar**: writing – review and editing, validation, supervision, conceptualization. **Vaishnavi rawat**: methodology, investigation, formal analysis, data curation. **Pooja Bargali**: visualization, writing – review and editing, validation, conceptualization. **Aashish Kumar Sagar**: methodology, investigation, formal analysis, data curation. **Stefania Garzoli**: formal analysis, writing – review and editing; **riddhiman lahiri**: methodology, investigation, formal analysis. **Shilpi Rawat**: writing – review and editing, methodology. **Avdhesh Kumar**: writing – review and editing, investigation, supervision. **Manmohan Singh Rawat**: visualization, validation. **Jolanta Maslowiecka**: Writing – review and editing. **Valery A. Isidorov**: writing – review and editing. **Dharmendra Singh Rawat**: writing – review and editing.

## Conflicts of Interest

The authors declare no conflicts of interest.

## Supporting information




**Supporting File 1**: cbdv71680‐sup‐0001‐SuppMat.docx
Table S1. Comparative Chemical Composition of Extracts of SGDE, SPDE, SADE, SATDE and STDE.

## Data Availability

Data will be made available on request.
