# Supplementary material for: GC–MS‐Based Phytochemical Profiling and Integrated Evaluation of Antioxidant, Antibacterial, and In Silico Studies of Kumaun Himalayan Strobilanthes Species
Source: Chem Biodivers. 2026 Sep 4;23(9):e71680. doi: 10.1002/cbdv.71680 (PMC13543764; doi:10.1002/cbdv.71680)
Supplement: Supplementary file 1 [file CBDV-23-e71680-s001.docx]

**Table S1.** Comparative Chemical Composition of Extracts of SGDE, SPDE, SADE, SATDE and STDE

| **S.No.** | **Compound** | **RI ^Calc^** | **RI^DB^** | **SGDE** | **SPDE** | **SADE** | **SATDE** | **STDE** |
| --- | --- | --- | --- | --- | --- | --- | --- | --- |
| 1. | Sclareol | 515-03-7 | 2218 | 2.69 | - | - | - | 10.72 |
| 2. | Sabinene | 972 | 974±2 | - | - | - | - | 0.01 |
| 3. | β-pinene | 977 | 979±2 | 0.34 | 0.08 | 0.01 | 0.01 | 0.01 |
| 4. | n-decane | 1000 | 1000 | - | - | - | 0.70 | - |
| 5. | p-cymene | 1023 | 1025±2 | 0.01 | 0.01 | 0.01 | 0.01 | 0.01 |
| 6. | Limonene | 1028 | 1030±2 | 0.18 | 0.01 | 0.01 | 0.01 | 0.01 |
| 7. | Eucaliptol | 1030 | 1032±2 | - | 0.12 | - | - | - |
| 8. | Eucalyptol | 1032 | 1032±2 | 0.27 | - | 0.01 | 0.01 | 0.01 |
| 9. | Linalool | 1101 | 1102 | - | 0.06 | - | - | 0.01 |
| 10. | *E*-2-octenoic acid | 1225 | 1227±18 | - | 0.17 | - | - | - |
| 11. | β-caryophyllene | 1417 | 1419±3 | 0.26 | - | - | - | - |
| 12. | *E*-2-decenoic acid | 1425 | - | - | 1.00 | - | - | - |
| 13. | Dihydroactinidiolide | 1534 | 1532±10 | - | 0.15 | - | - | - |
| 14. | Guaiol | 1600 | 1596±3 | - | 0.64 | 0.01 | 0.75 | - |
| 15. | *E*-2-dodecenoic acid | 1608 | - | - | 0.10 | - | - | - |
| 16. | β-azarone | 1627 | 1628±6 | - | - | - | - | 0.01 |
| 17. | Agarospirol | 1652 | 1643±3 | - | 0.29 | - | - | - |
| 18. | Loliolide | 1739 | - | - | 0.16 | - | - | - |
| 19. | Tetradecanoic acid | 1766 | 1768±5 | - | 0.39 | - | - | - |
| 20. | Dehydrovomifoliol | 1790 | 1800±N/A | 0.43 | 0.62 | - | 2.34 | 0.53 |
| 21. | 4,6-dimethoxy-2-benzofuran-1(3H)-one | 1819 | - | - | 0.93 | - | - | - |
| 22. | 6,9-dihydroxy-α-humulene | 1828 | 1828±N/A | - | - | 0.36 | - | - |
| 23. | Neophytadiene | 1831 | 1838±3 | 1.22 | 1.98 | 3.16 | 1.60 | 0.72 |
| 24. | 3,7,11,15-tetramethylhexadec-2-ene | 1835 | 1830±N/A | - | 0.29 | 0.45 | - | 0.01 |
| 25. | Hexafarnesylacetone | 1846 | 1844±4 | 1.23 | - | 1.36 | 0.47 | 0.52 |
| 26. | 8-hydroxyageraphorone | 1874 | 1865±N/A | - | 0.35 | - | - | - |
| 27. | 15,16-dinorlabd-12-ene, 8,13-epoxy- | 1879 | 1894±N/A | - | - | - | - | 0.01 |
| 28. | Palmitelaidic acid | 1944 | 1951±7 | - | 0.15 | - | - | - |
| 29. | Hexadecanoic acid | 1966 | 1962±N/A | 0.30 | 2.80 | 2.71 | 2.28 | - |
| 30. | Epimanool | 2053 | 2056±8 | 0.49 | - | - | - | 0.55 |
| 31. | Manool | 2063 | 2056±1 | - | - | - | - | 0.50 |
| 32. | Lycopodine | 2068 | - | 0.29 | - | - | - | - |
| 33. | Methyl lnoleate | 2094 | 2093±3 | - | 0.08 | - | - | - |
| 34. | Methyl linolenate | 2100 | 2099±3 | - | - | - | 3.41 | 1.72 |
| 35. | n-heneicosane | 2100 | 2100 | - | - | 0.49 | - | - |
| 36. | Methyl oleate | 2100 | 2095±9 | - | 0.15 | - | - | - |
| 37. | Linolenic acid | 2102 | 2139±N/A | 0.11 | - | 3.51 | 0.51 | 0.82 |
| 38. | Phytol | 2114 | 2114±6 | 8.12 | 1.86 | 3.21 | 2.88 | 5.81 |
| 39. | Coronorin E | 2121 | 2136±N/A | 0.43 | 0.13 | - | - | - |
| 40. | Linoleic acid | 2141 | 2139±N/A | - | 2.53 | 1.13 | 4.03 | - |
| 41. | Octadecanoic (stearic) acid | 2167 | 2172±8 | - | 0.70 | 0.62 | - | - |
| 42. | 2-methyldocosane | 2264 | 2263±2 | - | - | 0.65 | - | 0.28 |
| 43. | n-tricosane | 2300 | 2300 | 0.33 | 0.13 | 0.82 | 0.01 | 0.33 |
| 44. | 4,8,12,16-tetramethylheptadecan-4-olide | 2353 | 2364±N/A | - | 0.16 | 0.34 | - | - |
| 45. | n-tetracosane | 2400 | 2400 | - | - | - | - | - |
| 46. | Phytyl 2-methylbutanoate | 2434 | 2441±N/A | 1.96 | 0.16 | 0.25 | 0.01 | - |
| 47. | 2-methyltetracosane | 2464 | 2462±2 | 0.55 | - | 1.35 | - | 1.03 |
| 48. | n-pentacosane | 2500 | 2500 | 0.43 | 0.28 | 0.59 | - | 0.81 |
| 49. | Vilosin | 2541 | 2559±17 | 0.38 | - | - | - | - |
| 50. | Bis(2-ethylhexyl) phthalate | 2548 | 2535±16 | - | 0.83 | - | - | - |
| 51. | 3-methylpentacosane | 2573 | 2573±1 | - | - | 0.29 | - | - |
| 52. | Phytyl hexanoate | 2581 | 2568±N/A | 0.27 | 0.15 | 0.44 | - | - |
| 53. | n-hexacosane | 2600 | 2600 | 0.30 | 0.21 | 0.21 | - | - |
| 54. | 2-methylhexacosane | 2663 | - | - | - | 0.53 | - | 0.47 |
| 55. | 1-tetracosanol | 2695 | 2698±1 | 0.66 | - | - | - | 0.87 |
| 56. | n-heptacosane | 2700 | 2700 | 1.57 | 0.47 | 1.43 | 2.90 | 1.48 |
| 57. | 3-methylheptacosane | 2773 | 2773±1 | - | - | - | - | 0.22 |
| 58. | n-octacosane | 2800 | 2800 | 0.63 | 0.35 | 0.7 | 0.01 | 0.39 |
| 59. | Squalene | 2829 | 2827±8 | 2.19 | 1.01 | 2.23 | 2.57 | 2.51 |
| 60. | 2-methyloctacosane | 2863 | 2863±2 | 0.54 | 0.11 | 2.12 | - | 1.45 |
| 61. | n-nonacosane | 2900 | 2900 | 2.39 | 1.11 | 4.69 | 1.08 | 3.37 |
| 62. | 3-methylnonacosane | 2974 | 2973±1 | - | - | 0.85 | - | 0.37 |
| 63. | n-triacontane | 3000 | 3000 | 0.52 | 0.75 | 1.52 | 1.14 | 0.44 |
| 64. | β-tocopherol | 3034 | 3043±N/A | - | 0.09 | - | - | - |
| 65. | γ-tocopherol | 3049 | 3064±N/A | 0.78 | 0.82 | 0.74 | 1.36 | 0.49 |
| 66. | Tetracosahexaen-3-ol, 2,6,10,15,19,23-hexamethyl | 3054 | 3031±N/A | - | - | - | 0.92 | - |
| 67. | 2-methyltriacontane | 3063 | 3062±2 | 0.89 | - | 0.84 | 3.61 | 1.03 |
| 68. | n-hentriacontane | 3100 | 3100 | 3.70 | 1.95 | 5.80 | 1.40 | 4.22 |
| 69. | α-tocopherol | 3130 | 3131±N/A | 0.67 | 0.86 | 0.60 | 3.62 | 0.34 |
| 70. | Ergosta-5,7,9(11),22-tetraen-3-ol, (3β,22E)-516-85-8 | 3152 | 3182±32 | - | 0.16 | - | - | - |
| 71. | Ergosterol | 3190 | 3203±51 | - | 0.28 | - | - | - |
| 72. | n-dotriacontane | 3200 | 3200 | 0.70 | 0.50 | - | 0.01 | - |
| 73. | 24-methylenecholesterol | 3208 | - | - | - | 1.35 | - | - |
| 74. | 24-epicampesterol | 3215 | 2632±N/A | - | - | 2.23 | 7.89 | 2.27 |
| 75. | Cholestenone | 3233 | 3245±N/A | - | 0.14 | - | - | - |
| 76. | Stigmasterol | 3246 | 3249±N/A | 5.84 | 4.64 | 6.92 | 0.80 | 5.16 |
| 77. | 2-methyldotriacontane | 3262 | 3257±3 | 0.83 | - | 0.55 | - | 0.93 |
| 78. | Obtusifolio | 3281 | - | - | 0.16 | - | - | - |
| 79. | n-tritriacontane | 3300 | 3300 | 4.81 | - | - | 0.01 | 4.49 |
| 80. | β-sitosterol | 3311 | 3308±N/A | 11.28 | 10.10 | 11.29 | 1.48 | 8.17 |
| 81. | Isofucosterol | 3324 | 3343±N/A | 0.61 | 0.55 | - | - | - |
| 82. | β-amyrin | 3331 | 3336±N/A | 1.32 | 1.47 | 4.44 | 1.64 | 1.32 |
| 83. | β-amirone | 3356 | - | - | - | 0.46 | 5.34 | 0.01 |
| 84. | Lupen-3-one | 3361 | 3395±11 | - | 7.44 | - | - | - |
| 85. | Lupeol | 3380 | 3389±N/A | 5.85 | 22.63 | 9.07 | 1.34 | 5.74 |
| 86. | Glutin-5-en-3β-ol | 3391 | - | - | - | 1.81 | - | - |
| 87. | n-tetratriacontane | 3400 | 3400 | - | - | - | - | 0.31 |
| 88. | Lupenone | 3409 | 3406±N/A | - | - | 0.78 | - | - |
| 89. | 24-ethylenecycloartan-3-one | 3426 | - | - | 0.73 | - | - | - |
| 90. | Sitostenone | 3442 | 3447±12 | - | - | 0.98 | 0.76 | 0.46 |
| 91. | γ-sitostenone | 3444 | - | - | 4.24 | - | - | - |
| 92. | n-pentatriscontane | 3500 | 3500 | - | - | - | - | 0.32 |
| 93. | Fridelan-3-one | 3514 | 3533±22 | - | - | 1.39 | - | - |
| 94. | 2-triacontanone | 3528 | 3531±N/A | - | - | 0.35 | - | - |
| 95. | Phytyl palmitate | 3559 | 3550±N/A | - | - | 0.49 | - | - |
| 96. | 5α-stigmastane-3,6-dione | 3605 | 3601±N/A | - | 0.91 | - | - | - |
| 97. | Stigmastane-3,6-dione | 3606 | 3601±N/A | 0.43 | - | 0.34 | 1.47 | 0.43 |
| 98. | Betulin | 3730 | 3740±21 | - | 1.18 | 0.17 | - | 0.25 |
